# *Nigella sativa* and health outcomes: An overview of systematic reviews and meta-analyses

**DOI:** 10.3389/fnut.2023.1107750

**Published:** 2023-03-28

**Authors:** Zhongyu Li, Yang Wang, Qing Xu, Jinxin Ma, Xuan Li, Jiaxing Yan, Yibing Tian, Yandong Wen, Ting Chen

**Affiliations:** ^1^Department of Gastroenterology, Xiyuan Hospital, China Academy of Chinese Medical Sciences, Beijing, China; ^2^Department of Chinese Medicine, Eye Hospital, China Academy of Chinese Medical Sciences, Beijing, China

**Keywords:** *Nigella sativa*, health outcomes, meta-analysis, overview, systematic review

## Abstract

**Background:**

*Nigella sativa* (*N. sativa*) consumption has been associated with various health outcomes; however, the results are not completely consistent.

**Objectives:**

This overview of systematic reviews and meta-analyses aimed to evaluate the reporting and methodological quality, and to grade the available evidence of associations between *N. sativa* and health outcomes.

**Methods:**

PubMed, Cochrane Library, Embase, and Scopus databases were searched from their inception to September 30, 2022. The Preferred Reporting Items for Systematic Reviews and Meta-Analyses (PRISMA) 2009 statement, Assessment of Multiple Systematic Reviews (AMSTAR) 2 checklist, and Grades of Recommendations, Assessment, Development and Evaluations (GRADE) systems were used to assess the reporting, methodological, and evidence quality for each meta-analysis, respectively. The results were synthesized in a narrative form.

**Results:**

This overview included 20 eligible meta-analyses published in peer-reviewed journals between 2013 and 2021. The overall methodological quality was relatively poor, with only one moderate quality, four low quality, and 15 critically low quality studies. For reporting quality, items two, five, eight, nine, 15, and 24 need to improve. Among the 110 outcome indicators of the quality of evidence, five were graded as moderate, 17 as low, and 88 as very low. Risk of bias, inconsistency, and imprecision were the main downgrading factors.

**Conclusion:**

This overview suggests that *N. sativa* is beneficial for various clinical outcomes. However, there are certain limitations to reporting and methodological quality. The clinical efficacy of *N. sativa* requires confirmation in high-quality, large-sample, randomized controlled trials.

## 1. Introduction

*Nigella sativa (N. sativa)* is an annual flowering plant of the Ranunculaceae family that grows widely in Middle Eastern and European countries (1). It has been used as a functional food, health product and medicine for thousands of years, suggesting that it may have some potential benefits for people (2–4). In traditional medicine, *N. sativa* is used for respiratory, digestive, and cardiovascular diseases, such as asthma, dyspepsia, and hypertension, and to improve liver and kidney function (5–9). Many scientific studies have demonstrated that *N. sativa* has a broad spectrum of positive pharmacological effects, including antiviral (10), anti-inflammatory (11), hypotensive (12), hypoglycemic (13) and antitumor (14) effects. These biological properties are related to the abundance of several phytochemicals, including thymoquinone, terpenes, saponins, flavonoids, and essential oils (8, 15). These promising active ingredients and their biological properties make *N. sativa* a powerful natural candidate for the prevention and control of diseases. In recent years, several meta-analyses based on randomized controlled trials (RCTs) of *N. sativa* have assessed its association with health outcomes. However, no review articles have evaluated the scientific quality and summarized the reported outcomes. Consequently, guidance for clinical users and physicians is limited.

Overview is a novel method for assessing the scientific quality of published systematic reviews and meta-analyses in a specific domain (16, 17). This method has been applied in many medical fields including acupuncture (18, 19), saffron (20) and dietary interventions (21). However, despite the number of systematic reviews and meta-analyses that have evaluated the association between *N. sativa* supplementation and health outcomes, there are no comprehensive reviews to assess the reporting and methodological quality and summarize the evidence. Therefore, the purpose of this review is to provide practical information for patients and those responsible for making treatment decisions.

## 2. Methods

The current overview of meta-analyses is reported in accordance with the Preferred Reporting Items for Systematic Reviews and Meta-Analyses (PRISMA 2009) statement (22).

### 2.1. Search strategy

PubMed, Embase, Scopus, and Cochrane Library databases were searched from their inception to September 30, 2022. We used the following search strategies: (“*nigella sativa*” or “black cumin” or “black seed” or “black caraway” or “kalonji” or “thymoquinone”) and (“systematic review” or “meta-analysis”). No language restrictions were imposed. In addition, we manually screened the reference lists of the selected studies to identify additional studies that met the criteria. The full search strategy is listed in Supplementary Table S1. First, two independent reviewers (Y.W. and Q.X.) screened the records based on the titles and abstracts after duplicates were removed. The full texts of potentially eligible records were downloaded for further evaluation. Any disagreements were resolved by consulting a third reviewer (Y.D.W.).

### 2.2. Inclusion and exclusion criteria

Articles were eligible if they were meta-analyses conducted using systematic reviews. Details of the inclusion criteria were as follows: (1) population: adults aged ≥18 years, with no restrictions on sex or race; (2) intervention: oral *N. sativa* intervention with any dose and treatment duration; (3) comparator: placebo, no treatment, or conventional therapy; (4) outcomes: any health outcomes, for example, blood glucose, serum lipids, liver function, etc.; and (5) study design: meta-analyses of RCTs. To clarify the therapeutic effects of *N. sativa*, studies on multiherbal interventions were excluded. Non-human studies, original studies, conference abstracts, and letters were also excluded. In addition, we excluded studies administrated by the topical use or injection, as these formulations have different compositions and mechanisms.

### 2.3. Data extraction

Two researchers (J.X.M. and J.X.Y.) independently extracted data, including first author, year of publication, country, sample size, number of RCTs in the meta-analysis, intervention/comparation, risk of bias assessment, reported outcomes, and safety. Any disagreements were resolved by consulting a third reviewer (Y.D.W).

### 2.4. Assessing the quality of included studies

#### 2.4.1. Assessment of the reporting quality

We evaluated the reporting quality using the PRISMA 2009 statement (22). The PRISMA 2009 statement consists of 27 items in seven domains: title, abstract, introduction, methods, results, discussion, and funding. According to the reported completeness, each item was answered as “yes,” “partial yes,” or “no.”

#### 2.4.2. Assessment of methodological quality

We evaluated the methodological quality of the included studies using the Assessment of Multiple Systematic Reviews (AMSTAR) 2 checklist (23). The AMSTAR-2 checklist consists of 16 items, and each item could be answered as “yes,” “partial yes” or “no.” The overall methodological quality of each study was then classified as “high,” “moderate,” “low” or “critically low.”

#### 2.4.3. Grading the evidence quality

We used the Grading of Recommendations, Assessment, Development and Evaluation (GRADE) system to assess evidence quality (24), which includes five domains: risk of bias, inconsistency, indirectness, imprecision, and publication bias. And the quality of evidence for each outcome was graded as “high,” “moderate,” “low” and “very low.”

Two researchers (X. L. and Y. B. T.) independently assessed the reporting, methodological, and quality of evidence. Any disagreements were resolved by consulting a third researcher (T.C.).

### 2.5. Statistical analysis

The efficacy and safety results reported in the included meta-analyses with *N. sativa* were synthesized in a narrative review, including risk ratio (RR), odds ratio (OR), mean difference (MD), weighted mean difference (WMD), and standard mean difference (SMD), along with *P*-value and a 95% confidence interval (CI). I^2^ was used to test for heterogeneity. In addition, we calculated compliance rates for PRISMA 2009 statement and AMSTAR 2 checklist item in meta-analyses, and reported the number and percentage of “yes,” “partial yes” or “no” responses. The reporting and methodological quality were visualized using a radar plot and bar chart, respectively. According to a previous study, a percentage of “yes” < 60% for an item indicates a need for improvement (25). Excel 2016 (Microsoft Corporation, WA, USA) was used for data analysis and visualization.

## 3. Results

### 3.1. Literature search results

**Figure 1:**
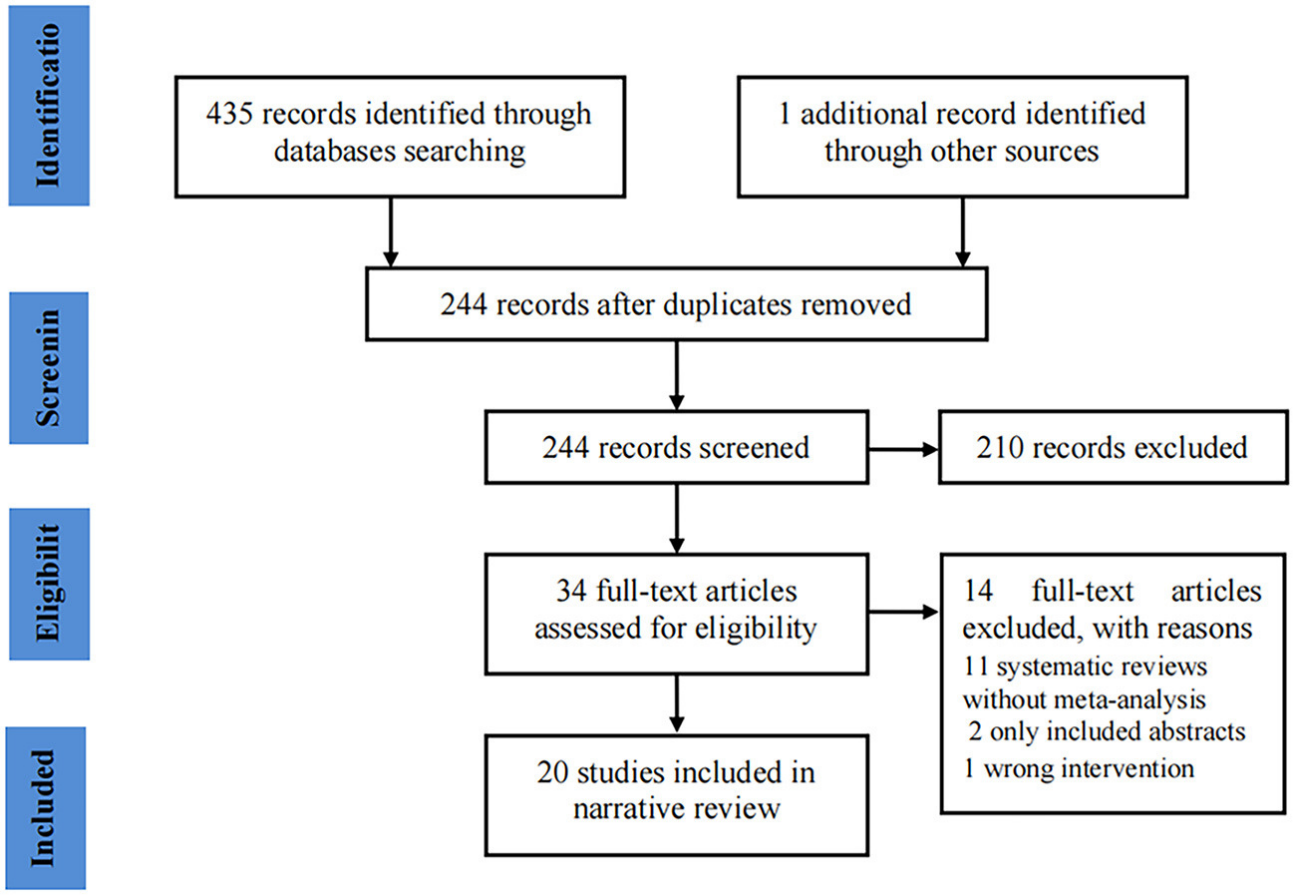
PRISMA flow chart for the included studies.

Our initial search identified 436 potential records. After removing duplicates, a total of 244 records remained. Subsequently, 210 records were excluded after screening titles and abstracts. The full texts of 34 records were further evaluated, and 20 records (26–45) were eventually included in the analysis (Figure 1 shows the flow chart of study selection).

### 3.2. Basic characteristics of the included literature

All meta-analyses were published in peer-reviewed journals between 2016 and 2022. These studies were conducted in seven regions: 13 from Iran (28, 30, 33, 34, 37–45), two from China (32, 35), and one each from Australia (26), India (27), United Kingdom (29), the USA (31), and Indonesia (36). The number of RCTs ranged from three to 50, with 187–3,679 subjects. Most studies have reported forms of *N. sativa*, including capsules, oils, and powders. Nineteen studies reported doses of *N. sativa*, ranging from 0.5 to 6 g or 2.5 to 5 ml daily (26–30, 32–45). Five studies reported the frequency of *N. sativa* administration, which varied between once, twice, and thrice a day (29, 32–35). The treatment duration ranged from 2 weeks to 1 year. Four studies registered protocols on the PROSER platform (27–29, 39) and one in the Cochrane library (31). In terms of the risk of bias tools, 10 studies used the Cochrane risk of bias tool (26–28, 30, 31, 33, 35, 39, 44, 45), nine studies used the Jadad scale (32, 34, 36–38, 40–43), and one used the standardized JBI critical appraisal checklist (29). Table 1 summarizes the basic characteristics of the included meta-analyses.

**Table 1:** Characteristics of the included studies.

**Reference**	**Country**	**Health status**	**Interventions/ comparations**	**Number of primary studies**	**Sample size (I/C)**	**Dose**	**Frequency**	**Form**	**Duration**	**Registration** ** information**	**Bias of risk assessment**	**Reported outcomes**	**Safety**
Saeede Saadati (26)	Australia	Prediabetes and T2DM	*N*. sativa */*Placebo, routine therapies	11	666 (338/328)	0.9 to 5 g/day	NR	Oil = 9; Ext = 2	2–6 months	No	Cochrane	BMI, FPG, OGTT, HbA1c, fasting insulin, HOMA-IR, TG, TC, LDL-C, HDL-C, CRP, and MDA	No
Anoop Tiwari (27)	India	NAFLD	*N*. sativa */* Placebo	4	224 (NR)	1,000 to 2,000 mg/day	NR	Cap = 2; Oil = 2	8–12 weeks	PROSPERO (CRD42020179378)	Cochrane	ALT, AST, TG, LDL-C, HDL-C, BMI	No
Sahar Golpour-hamedani (28)	Iran	Adults (Dyslipidemia = 2; Mets = 4; Obesity = 3; Hypertension = 4; Healthy volunteer = 4; Menopausal women = 1; PCOS = 1; T2DM = 1; Cardiovascular diseases = 1; NAFLD = 1)	*N*. sativa */* Placebo, standard therapy	22	1208	Pow: 500 to 1,000 mg/day; Cap: 400 to 2,000 mg/day; Oil: 3 to 5 ml/day	NR	Pow = 5; Cap = 12; Oil = 4; NR = 1	3 weeks to 1 year	PROSPERO (CRD42022315493)	Cochrane	SBP, DBP	No
Kaushik Chattopadhyay, (29)	United Kingdom	T2DM	*N*. sativ*a/* Placebo, conventional therapy, no treatment	8	NR	Cap: 0.5 to 3g/day; Oil: 5 ml/day	Qd = 1; Bid = 5; TID = 2	Cap = 7; Oil = 1	8–13 weeks	PROSPERO (CRD42018118285)	Standardized JBI critical appraisal checklist	FPG, PPBG, HbA1c, fasting insulin, HOMA-IR, BMI, BW, TG, TC, LDL-C, HDL-C	Yes
Neda Azizi (30)	Iran	Adults (NAFLD = 4; T2DM = 1; Postmenopausal women with osteoporosis = 1; obese = 1; hypercholesterolemia = 1)	*N*. sativa/Placebo	8	519 (281/279)	Oil:2.5 to 3 ml; Cap:0.5 to 1 g	Bid = 1; Tid = 1; NR = 6	Cap = 4; Oil = 4	6–12 weeks	No	Cochrane	AST, ALT	No
Dinesh Gyawali (31)	USA	Hypercholesterolemia	*N*. sativa/Placebo	3	NR	NR	NR	NR	4–8 weeks	Cochrane Database	Cochrane	TC, TG, HDL, LDL	No
Anqiang Han (32)	China	Asthma	*N*. sativa/Placebo	4	187 (NR)	0.5–1 g	Bid=1; NR=3	Cap=3; Ext=1	4 weeks to 3 months	No	Jadad scale	ACT, FEV_1_, PEF, IL-4, IFN-γ	No
Sanaz Malekian (33)	Iran	Adults (Obesity = 3; RA = 2; T2DM = 2; Mets = 1; UC = 1; NAFLD = 2)	*N*. sativa/Placebo	11	710 (NR)	0.5–3 g/day	Bid=3; Tid=3; Qd=2; Qid=3	Cap=11	6 weeks to 1 year	No	Cochrane	TNF-α, hs-CRP, IL-6, SOD, TAC, MDA	Yes
Rahele Sadat Montazeri (34)	Iran	Adults (Obesity = 2; RA = 2; T2DM = 2; Mets = 2; UC = 1; NAFLD = 1)	*N*. sativa/Placebo	10	630 (NR)	1–3g/day	NR	NR	6–48 weeks	No	Jadad scale	hs-CRP, TNF-α, MDA, TAC, SOD	NR
Gang Tang (35)	China	NAFLD	*N*. sativa/Placebo	5	358 (179/179)	Cap: 75 mg to 2 g/day; Oil: 5 ml/day	Bid = 2; Tid = 1; NR = 2	Oli = 2; Cap = 3	8–24 weeks	No	Cochrane	ALT, AST, insulin, FBS, TC, TG, HDL, LDL, hs-CRP, TNF-α, grade of fatty liver	Yes
M.Ardiana (36)	Indonesia	Adults (Obese = 1; RA = 1; T2DM = 2; UC = 1)	*N*. sativa/Placebo	5	293 (152/141)	500 mg−3g/day	NR	Cap = 5	8–48 weeks	No	Jadad scale	MDA, SOD, TAC	NR
Jamal Hallajzadeh (37)	Iran	Adults (T2DM = 12; Mild hypertension = 2; Insulin resistance = 1; Overweight or obesity = 7; Mets = 9; RA = 2; UC = 1; NAFLD = 4; Dyslipidemia = 6; Healthy volunteer = 4; Menopausal women = 2; HT = 1; kidney disease = 1; other disease = 5)	*N*. sativa /Placebo	50	3,679 (1,932/ 1,747)	Cap: 400–3,000 mg/day; Oil: 2.5–5 ml/day; Pow: 400 to 3,000 mg/day; Ext: 700 mg/day	NR	Oil = 19; Pow = 11; Cap = 13; Ext = 2; NR = 12	2–12 months	No	Jadad scale	TC, TG, LDL-C, VLDL-C, HDL-C, FBS, HbA1C, Insulin, HOMA-IR, CRP, TNF-α, MDA, TAC	NR
Mohsen Mohit (38)	Iran	Adults (T2DM = 3; RA = 2; NAFLD = 2; UC = 1; Obese = 2; HT = 1; Helicobacter infected patients = 1)	*N*. sativa/Placebo	12	659 (339/320)	1–3 g/day	NR	Cap = 10; Pow = 2	6–48 weeks	No	Jadad scale	TAC, MDA, TNF-α, IL-6, CRP	NR
Elham Razmpoosh (39)	Iran	Adults (Stable angina = 1; Hypertension = 2; Kidney disease = 2; T2DM = 2; Obese = 3; Liver diseases = 2; Healthy individuals = 4; Osteoporosis = 1; Hypercholesterolemia = 1; Menopause women = 1)	*N*. sativa or black seed family/Placebo	19	1,295	0.5–6 g/day	NR	Oil = 12; Pow = 7	4–42 weeks	PROSPERO (CRD42018102229)	Cochrane	ALT, AST, ALP, BUN, CREA, uric acid, bilirubin, urine, serum total protein, albumin	NR
Rahele Tavakoly (40)	Iran	Adults (Mets = 2; Obese = 2; RA = 1; UC = 1; NAFLD = 1)	*N*. sativa/Placebo	7	439 (222/217)	1–3 g/day	NR	Oil = 2; Pow = 5	6–12 weeks	No	Jadad scale	Serum CRP	NR
Seyed Mohammad Mousavi (41)	Iran	Adults (T2DM = 2; Hypertension = 1; Hypercholesterolemia = 1; HT = 1; NAFLD = 1; Obesity and overweight = 3; Healthy subjects = 4)	*N*. sativa/Placebo	13	875 (445/430)	Oil: 5 ml/day or 3 g/day; Cap: 100 mg to 2 g/day	NR	Oil = 4; Cap = 9	6–13 weeks	No	Jadad Score	BW, BMI, WC	NR
Nazli Namazi (42)	Iran	Adults (Overweight/obesity = 3; Diabetes = 2; Mets = 2; Hypertension = 1; RA = 1; Healthy subjects = 2)	*N*. sativa/Placebo	11	783(NR)	Pow: 1 to 2 g/day; Oil: 3 to 5g/day; Ext: 100 to 200mg/day	NR	Pow = 5; oil = 5; Ext = 1	6–12 weeks	No	Jadad scale	BW, BMI, WC	Yes
Reza Daryabeygi-Khotbehsara (43)	Iran	T2DM	*N*. sativa/Placebo or standard treatment	7	555 (255/250)	0.5 to 2g/day	NR	Pow = 4; Oil = 3	2 to 12 months	No	NO	FBS, HbA1c, TC, TG, HDL, LDL	NR
Amirhossein Sahebkar (44)	Iran	Adults (Mets = 2; Overweight/obesity = 3; Hypertension = 1; Hyperlipidemia = 5; T2DM = 3; Menopausal women = 3; Healthy subjects = 2)	*N*. sativa/Placebo	17	1185 (619/569)	Pow: 1 to 2 g/day; Oil: 100 mg to 3g/day or 5 ml/day	NR	Pow = 10; Oil = 7	4 weeks to 3months	No	Cochrane	TC, LDL-C, HDL-C, TG	NR
Amirhossein Sahebkar (45)	Iran	Adults (Healthy subjects = 3; Mets = 2; Hypertension = 1; Hypercholesterolemia = 1; Menopausal women = 1; Perimenopausal women = 1; Obese = 2)	*N*. sativa/Placebo or standard treatment	11	860 (435/425)	Pow: 500 mg to 2 g/day; Oil: 200 mg to 3 g/day or 5 ml/day	NR	Pow = 8; Oil = 3	4–12 weeks	No	Cochrane	BP, DBP	Yes

T2DM, type 2 diabetes mellitus; Mets, metabolic syndrome; NAFLD, non-alcoholic fatty liver diseases; RA, arthritis rheumatoid; Mets, metabolic syndrome; UC, ulcerative colitis; HT, Hashimoto's thyroiditis; pow, Power; ext, extract; cap, capsule; BMI, Body Mass Index; FPG, fasting plasma glucose; OGTT, oral glucose tolerance test; HbA1c, hemoglobin A1c; HOMA-IR, homeostatic model assessment of insulin resistance; TG, triglyceride; TC, total cholesterol; LDL-C, low-density lipoprotein cholesterol; HDL-C, high-density lipoprotein cholesterol; CRP, C-reactive protein; CREA, Creatinine; MDA, malondialdehyde; ALT, alanine aminotransferase; AST, aspartate aminotransferase; ALP, alkaline phosphatase; FBG, fasting blood glucose; PPBG, postprandial blood glucose; BW, body weight; ACT, asthma control test; FEV1, forced expiratory volume at 1s; PEF, peak expiratory flow; IL-4, interlukin-4; IFN-γ, interferon γ; TNF-α, tumor necrosis factor-α; hs-CRP, high-sensitive C-Reactive Protein; IL-6, interlukin-6; SOD, superoxide dismutase; TAC total antioxidant capacity; VLDL, very low-density lipoproteins; BUN, blood urea nitrogen; SBP, systolic blood pressure; DBP, diastolic blood pressure.

### 3.3. Results of reporting quality

**Figure 2:**
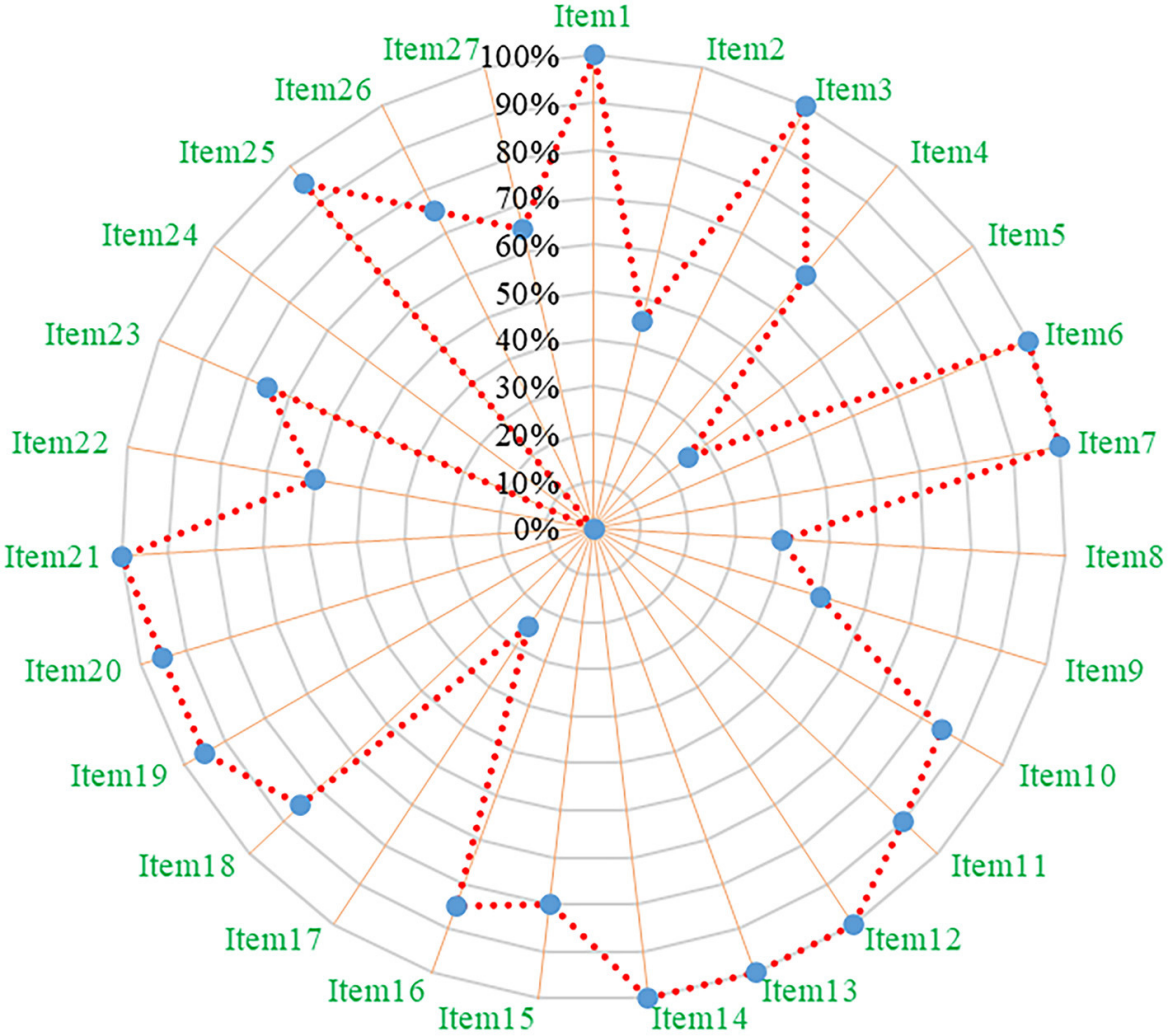
Reporting quality of included meta-analyses based on the PRISMA 2009 statement.

According to the PRISMA 2009 statement, 20 of the 27 items had a “yes” response rate of more than 60%, indicating that the included meta-analyses contained relatively complete reporting quality. However, there were limitations related to the following items: item two (abstract: structured abstract), five (method: protocol and registration), eight (method: search), nine (method: study selection), 17 (result: study selection), 22 (result: risk of bias across studies), and 24 (discussion: summary evidence) (see Figure 2 and Supplementary Table S2).

### 3.4. Results of methodological quality

**Figure 3:**
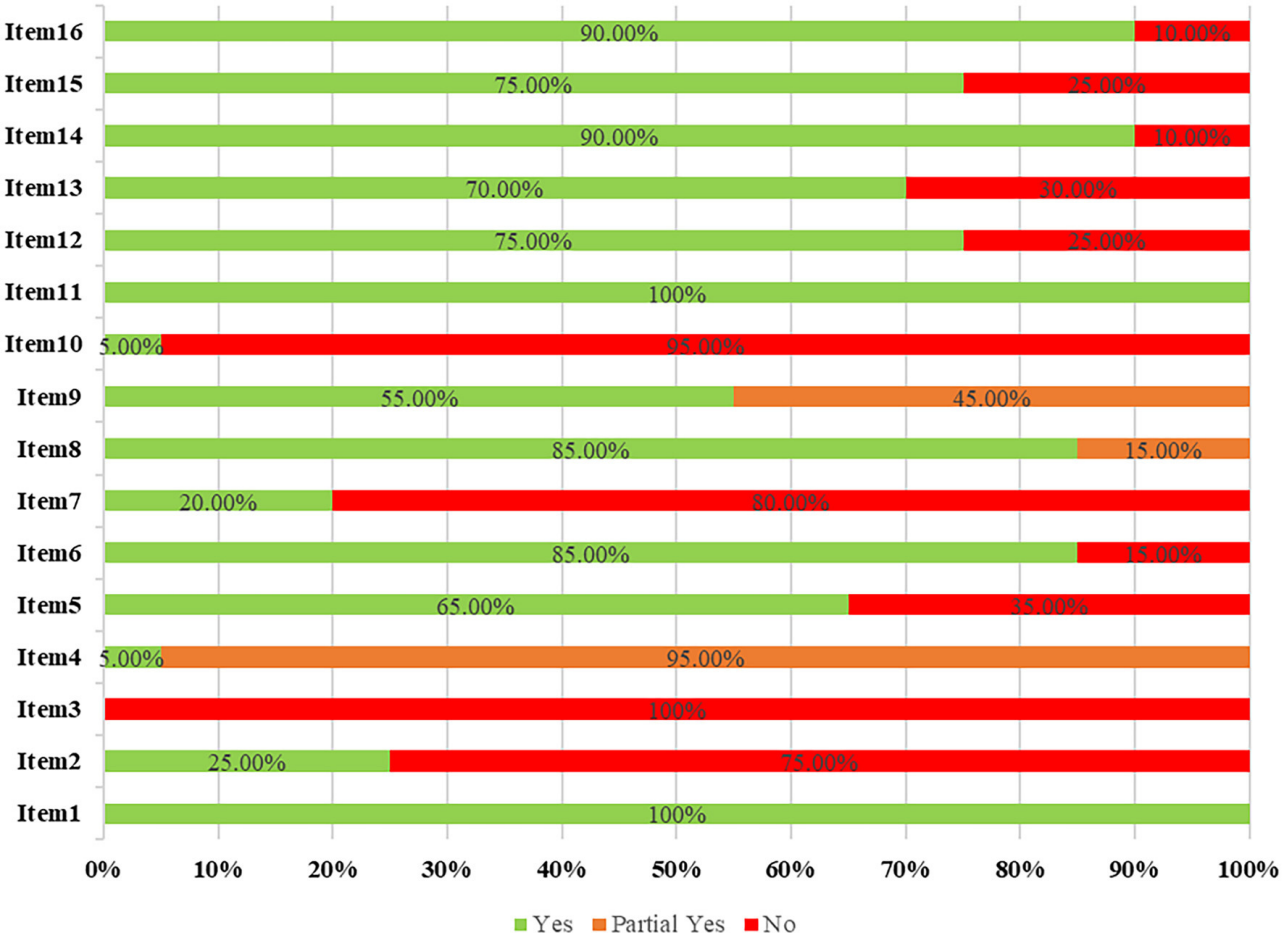
Methodological quality of included meta-analyses based on the AMSTAR-2 checklist.

The results of the overall methodological quality evaluated using the AMSTAR-2 checklist revealed that only one study was of moderate quality, four studies were of low quality, and the other 15 meta-analyses were of critically low quality (Supplementary Table S3). Methodological quality limitations included the following items: item two (register protocol prior to conducting the review), three (explain selection of the study designs in the review), seven (provide a list of excluded studies and justify the exclusions), and 10 (report the source of funding for the individual studies) (see Figure 3 and Supplementary Table S3).

### 3.5. Results of the quality of evidence

There were 110 outcome indicators in 20 meta-analyses. Five were graded as moderate-quality, 17 as low-quality, and 88 as very-low-quality evidence. However, there is no high-quality evidence for this. The evidence was mostly downgraded owing to the risk of bias, inconsistency, and imprecision (Supplementary Table S4).

### 3.6. Therapeutic effect of *N. sativa* on health outcomes

#### 3.6.1. Blood glucose and insulin secretion

Four studies evaluated the effects of *N. sativa* on glucose metabolism and insulin function (26, 29, 37, 43). All studies found that *N. sativa* reduced hemoglobin A1c (26, 29, 37, 43). Three studies observed that the consumption of *N. sativa* reduced fasting plasma glucose (FPG) levels (26, 37, 43), but one study found *N. sativa* had no effect on FPG in type-2 diabetes (29). In addition, *N. sativa* had no significant effect on the oral glucose tolerance test (OGTT) (26), fasting insulin levels (26, 29), homeostasis model assessment of insulin resistance (HOMA-IR) (26, 29, 37) and postprandial blood glucose (PPBG) (29).

#### 3.6.2. Serum lipids

Eight studies evaluated the effects of *N. sativa* on lipid profiles (26, 27, 29, 31, 35, 37, 43, 45). Six studies found that *N. sativa* reduced total cholesterol (TC) in patients with metabolic disorders (26, 29, 31, 37, 43, 45), but one study showed that *N. sativa* had no effect on patients with non-alcoholic fatty liver disease (NAFLD) (35). Seven studies found that *N. sativa* significantly reduced triglyceride (TG) levels (27, 29, 31, 37, 45), although four studies revealed that it did not change TG levels (26, 31, 35, 43). Six studies found that *N. sativa* reduced low-density lipoprotein cholesterol (LDL-C) levels (26, 27, 29, 37, 43, 45), drawing consistent conclusions. Only one study showed a significant effect on high-density lipoprotein cholesterol (HDL-C) levels (27), whereas the remaining five studies found that *N. sativa* did not change HDL-C levels (26, 29, 31, 43, 45).

#### 3.6.3. Blood pressure

Two studies evaluated the effects of *N. sativa* on blood pressure (28, 45). The results showed that *N. sativa* significantly reduced systolic and diastolic blood pressure in adults (28, 45).

#### 3.6.4. Body composition

Five studies evaluated the effects of *N. sativa* on body parameters (26, 27, 29, 41, 42). Three studies reported that *N. sativa* significantly reduced body weight (BW) (29, 41, 42). Two studies observed that *N. sativa* significantly reduced the body mass index (BMI) (41, 42), however, three studies found no effect on BMI (26, 27, 29). One study showed that *N. sativa* reduced waist circumference (WC) (42), but another showed no effect on WC (41).

#### 3.6.5. Inflammatory markers

Eight studies evaluated the effects of *N. sativa* on inflammatory markers (26, 32–35, 37, 38, 40). In adults, studies have observed that *N. sativa* intake significantly reduced tumor necrosis factor-α (TNF-α) (26, 33, 34), high-sensitivity C-reactive protein (hs-CRP) (34, 35), interleukin-6 (IL-6) (33), and C-reactive protein (CRP) (38, 40). However, other meta-analyses have shown that *N. sativa* supplementation had no effect on TNF-α (37, 38), hs-CRP (33), IL-6 (33), and CRP (37). In addition, one study observed that consumption *N*. sativa decreased interleukin-4 and increased interferon-γ in patients with asthma, but the difference was not statistically significant (32).

#### 3.6.6. Oxidative stress factors

Six studies evaluated the effects of *N. sativa* on oxidative stress factors (26, 33, 34, 36–38). However, these results were contradictory. In adults, studies have shown that *N. sativa* reduced malondialdehyde (MDA) (26, 34, 38) and increased superoxide dismutase (SOD) (33, 34, 36) and total antioxidant capacity (TAC) (33, 34, 38). The remaining meta-analyses found that *N. sativa* had no significant effects on MDA (33, 36, 37) and TAC (36, 37).

#### 3.6.7. Asthma

One study evaluated the effects of *N. sativa* on asthma (32). The results showed that *N. sativa* supplementation improved asthma control test scores and forced expiratory volume at 1s in patients with asthma, however, it had no significant effect on peak expiratory flow (32).

#### 3.6.8. Liver and kidney parameters

Four studies evaluated the effects of *N. sativa* on liver parameters (27, 30, 35, 39) and one study evaluated kidney parameters (30). One study found that *N. sativa* significantly improves fatty liver grading in patients with NAFLD (35). Three studies reported that *N. sativa* reduced aspartate aminotransferase (AST) levels (27, 30, 35). However, one study reported that *N. sativa* failed to reduce AST levels (39). Two studies found *N. sativa* reduced alanine aminotransferase (ALT) levels (27, 35), whereas two other studies found *N. sativa* had no effect on ALT levels (30, 39). In addition, one study observed that the use of *N. sativa* significantly reduced the alkaline phosphatase levels (39). In terms of kidney parameters, *N. sativa* significantly reduced urea nitrogen, but had no effect on creatinine, bilirubin, and uric acid levels (30).

#### 3.6.9. Safety

Five meta-analyses reported adverse events (29, 33, 35, 42, 45). The main adverse events were digestive symptoms such as stomach pain, diarrhea, nausea, and vomiting, as well as weakness and weight loss. However, no study has reported serious adverse events.

## 4. Discussion

In recent years, plant-based foods and herbs as therapeutic alternatives, have received a great deal of attention from both researchers and the general public (46, 47). Medicinal plants can be used for the treatment and prevention of various non-communicable diseases because they contain a wide range of bioactive phytochemicals and have different metabolic effects (48, 49). This is the first overview to synthesize the available meta-analyses for *N. sativa* and evaluate the reporting, methodological, and evidence quality. We found that *N. sativa* has a variety of potential effects on different indicators in clinical practice, including blood glucose, inflammatory markers, oxidative stress factors, serum lipids, blood pressure, liver and kidney parameters, and even asthma indicators. The therapeutic effects suggested that *N. sativa* has beneficial effects in various diseases and may be a promising complementary and alternative therapy.

The overall reporting quality of the meta-analyses was poor, highlighting the importance of future reviews to improve the standards. Regarding the PRISMA 2009 statement, items two, five, eight, nine, 17, 22, and 24 need significant improvement. In recent years, the problem of reproducibility and the waste of resources in biomedical research have caused considerable concern in the scientific community (50, 51). However, comprehensive and transparent reporting of the study design, study process, and final outcomes is key to avoiding these problems. Therefore, we strongly recommend that future meta-analyses of *N. sativa* should be performed in accordance with the PRISMA statement.

In terms of methodological quality, we found that items two, three, seven and 10 should be improved based on the AMSTAR-2 checklist. The registration of protocols can improve the transparency and help avoid the potential risk of bias (52, 53). It can also reduce duplicate work between different research groups and continue studies to date (54). The authors are encouraged to register their protocols in free and open databases, such as the PROSPERO platform and Cochrane Library, to avoid study bias (55). The AMSTAR-2 checklist requires review authors to explain why they chose a particular study design for meta-analysis (23), as systematic reviews should be “comprehensive” and specific study designs should be selected for different purposes. Furthermore, a comprehensive literature search strategy is the basis and a guarantee of meta-analysis. This helps to avoid missing data, leading to selective bias and obtaining the correct conclusions (56). In addition, the authors are required provide a full list of excluded studies and justify their exclusions, which can help the readers judge the extent of study inclusion and the accuracy of manuscript selection. Finally, the authors should clearly report a statement about funding sources and conflicts of interest. This can help other researchers judge the reliability of the conclusion and prevent bias that might favor funders (57). For example, authors may present favorable results and/or exaggerate the effects of drugs provided by industry funders (58). Therefore, the use of rigorous methodology reduces the risk of bias and improves the reliability of the conclusions.

The findings from the included meta-analyses suggested that *N. sativa* has potential efficacy in treating various diseases. However, according to the GRADE system, we found only five moderate quality, 17 low quality, and 88 very low quality studies, with an overall poor quality of evidence. The highest downgrading factor was the risk of bias. This was mainly due to RCTs with unclear or missing randomization, blinding, and allocation concealment. Therefore, in future studies, designers should pay more attention to the design and implementation processes. Another downgrading factor was inconsistency, with most studies showing high heterogeneity (*I*^2^ > 50%). This may be related to the different subjects, multiple forms of *N. sativa* supplements, and treatment duration. Future meta-analyses should explore potential heterogeneity based on subgroup, meta-regression, and sensitivity analyses. It is important for researchers to report in detail on the bioactive constituents of *N. sativa* and to transparently report on the *N. sativa* species, the dose and frequency of intervention administration, treatment duration, and adherence. Although most meta-analyses have provided certainty about the clinical efficacy of *N. sativa*, the overall sample size was low, suggesting that there remains a need for clinical evidence from high-quality, large-sample RCTs.

### 4.1. Strengths and limitations

This overview has several strengths and limitations. Regarding the strengths, this is the first overview to comprehensively summarize the clinical evidence of *N. sativa* supplementation and provide visualization of reporting and methodological quality. The results make up the knowledge gap regarding *N. sativa* supplements and can be used to guide further research and clinical decision-making. This study has several limitations. First, this overview only used a descriptive method, making it difficult to evaluate primary studies. Second, although two researchers who have been trained and passed the pre-test, independently conducted literature screening and quality evaluation, subjective factors cannot be eliminated and may affect objectivity. Third, because most studies did not mention adverse events, it is difficult to accurately assess the safety of *N. sativa* in clinical practice.

## 5. Conclusion

This overview suggests that *N. sativa* has the potential to improve different clinical outcomes, such as blood glucose, inflammatory markers, oxidative stress factors, serum lipids, blood pressure, liver and kidney parameters, and even asthma indicators. However, there are certain limitations in reporting and methodological quality, and future studies should improve the administration process. In addition, the clinical efficacy of *N. sativa* needs to be confirmed in high-quality, large-sample RCTs to generate more evidence-based clinical practice.

## Data availability statement

The original contributions presented in the study are included in the article/Supplementary material, further inquiries can be directed to the corresponding author.

## Author contributions

ZL designed and drafted the manuscript. YW, QX, JM, JY, XL, and YT performed the literature search, extracted the data, and assessed their quality. ZL, TC, and YW analyzed and interpreted the data. All the authors participated in this study and reviewed and agreed to publish this article.
