# Supplementary material for: Nigella sativa and health outcomes: An overview of systematic reviews and meta-analyses
Source: Front Nutr. 2023 Mar 28;10:1107750. doi: 10.3389/fnut.2023.1107750 (PMC10086143; doi:10.3389/fnut.2023.1107750)
Supplement: Supplementary file 1 [file Table_1.DOCX]

Supplementary Material

# Supplementary Table S1. Search terms used across the various databases

| PubMed  59 | ((“Nigella sativa”[Mesh]) OR ((((((Black cumin[Title/Abstract]) OR (Nigella sativa[Title/Abstract])) OR (black seed[Title/Abstract])) OR (black caraway[Title/Abstract])) OR (kalonji[Title/Abstract])) OR (thymoquinone[Title/Abstract]))) AND ((((((Meta-analysis[Title/Abstract]) OR (Systematic review[Title/Abstract])) OR (Meta analyses[Title/Abstract])) OR (meta analysis[Title/Abstract])) OR (Systematic reviews[Title/Abstract])) OR ((“Meta-Analysis” [Publication Type] OR “Meta-Analysis as Topic”[Mesh]) OR (“Systematic Review” [Publication Type] OR “Systematic Reviews as Topic”[Mesh]))) |
| --- | --- |
| Scopus  204 | ( TITLE-ABS-KEY ( “black cumin” ) OR TITLE-ABS-KEY ( “nigella sativa” ) OR TITLE-ABS-KEY ( “black seed” ) OR TITLE-ABS-KEY ( “black caraway” ) OR TITLE-ABS-KEY ( “kalonji” ) OR TITLE-ABS-KEY ( “thymoquinone” ) ) AND ( TITLE-ABS-KEY ( “meta-analysis” ) OR TITLE-ABS-KEY ( “meta analyses” ) OR TITLE-ABS-KEY ( “systematic review” ) OR TITLE-ABS-KEY ( “systematic reviews” ) OR TITLE-ABS-KEY ( “meta analysis” ) ) |
| Embase  172 | (‘black cumin’/exp OR ‘nigella sativa’: ti, ab, kw OR ‘black cumin’: ti, ab, kw OR ‘black seed’: ti, ab, kw OR ‘black caraway’: ti, ab, kw OR ‘kalonji’: ti, ab, kw OR ‘thymoquinone’: ti, ab, kw) AND (‘meta analysis’/exp OR ‘systematic review’/exp OR ‘systematic review’: ti, ab, kw OR ‘meta analyses’: ti, ab, kw OR ‘meta analysis’: ti, ab, kw OR ‘systematic reviews’: ti, ab, kw) |
| Cochrane  0 | ((MeSH descriptor: [Nigella sativa] explode all trees) OR (Nigella sativa): ti, ab, kw OR (Black cumin): ti, ab, kw OR (black caraway):ti, ab, kw OR (kalonji): ti, ab, kw OR (thymoquinone): ti, ab, kw (Word variations have been searched) in Cochrane Reviews) in Cochrane Reviews |
