# Supplementary material for: Nigella sativa and health outcomes: An overview of systematic reviews and meta-analyses
Source: Front Nutr. 2023 Mar 28;10:1107750. doi: 10.3389/fnut.2023.1107750 (PMC10086143; doi:10.3389/fnut.2023.1107750)
Supplement: Supplementary file 2 [file Table_2.DOCX]

Supplementary Material

**Supplementary Table S2 Results of the PRISMA 2009 statement.**

| **Reference** | **Item 1** | **Item 2** | **Item 3** | **Item 4** | **Item 5** | **Item 6** | **Item 7** | **Item 8** | **Item 9** | **Item 10** | **Item 11** | **Item 12** | **Item 13** | **Item 14** | **Item 15** | **Item 16** | **Item 17** | **Item 18** | **Item 19** | **Item 20** | **Item 21** | **Item 22** | **Item 23** | **Item 24** | **Item 25** | **Item 26** | **Item 27** |
| --- | --- | --- | --- | --- | --- | --- | --- | --- | --- | --- | --- | --- | --- | --- | --- | --- | --- | --- | --- | --- | --- | --- | --- | --- | --- | --- | --- |
| [Saeede Saadati](https://pubmed.ncbi.nlm.nih.gov/?size=50&term=Saadati+S&cauthor_id=36034891)，2022(Saadati et al., 2022) | Y | P | Y | Y | N | Y | Y | Y | Y | Y | Y | Y | Y | Y | Y | Y | N | Y | Y | Y | Y | N | Y | P | Y | Y | Y |
| Anoop Tiwari，2022(Tiwari et al., 2022) | Y | P | Y | Y | Y | Y | Y | N | Y | N | Y | Y | Y | Y | N | Y | N | Y | Y | Y | Y | N | Y | P | Y | Y | Y |
| Sahar Golpour hamedani, 2022(Golpour-Hamedani et al., 2022) | Y | Y | Y | P | Y | Y | Y | Y | Y | Y | Y | Y | Y | Y | Y | Y | Y | Y | Y | Y | Y | Y | Y | P | Y | Y | Y |
| Kaushik Chattopadhyay, 2022(Chattopadhyay et al., 2022) | Y | Y | Y | P | Y | Y | Y | Y | Y | Y | Y | Y | Y | Y | Y | Y | Y | Y | P | N | Y | N | N | Y | Y | Y | Y |
| [Neda Azizi](https://pubmed.ncbi.nlm.nih.gov/?sort=pubdate&size=50&term=Azizi+N&cauthor_id=33564654)，2021(Azizi et al., 2021) | Y | P | Y | P | N | Y | Y | Y | N | Y | Y | Y | Y | Y | N | Y | N | Y | Y | Y | Y | N | Y | P | Y | P | N |
| [Dinesh Gyawali](https://pubmed.ncbi.nlm.nih.gov/?sort=pubdate&size=50&term=Gyawali+D&cauthor_id=34071454)，2021(Gyawali et al., 2021) | Y | Y | Y | P | Y | Y | Y | Y | Y | N | N | Y | Y | Y | N | Y | N | Y | Y | Y | Y | N | N | P | P | Y | Y |
| [Anqiang Han](https://pubmed.ncbi.nlm.nih.gov/?sort=pubdate&size=50&term=Han+A&cauthor_id=34658694)，2021(Han and Shi, 2021) | Y | P | Y | N | N | Y | Y | N | N | Y | Y | Y | Y | Y | Y | Y | N | P | Y | Y | Y | N | N | P | Y | P | N |
| [Sanaz Malekian](https://pubmed.ncbi.nlm.nih.gov/?sort=pubdate&size=50&term=Malekian+S&cauthor_id=34187123)，2021(Malekian et al., 2021) | Y | P | Y | P | N | Y | Y | N | Y | Y | Y | Y | Y | Y | N | N | N | Y | Y | Y | Y | N | N | P | Y | Y | Y |
| [Rahele Sadat Montazeri](https://pubmed.ncbi.nlm.nih.gov/?sort=pubdate&size=50&term=Montazeri+RS&cauthor_id=33559935)，2021(Montazeri et al., 2021) | Y | P | Y | Y | N | Y | Y | N | P | Y | Y | Y | Y | Y | Y | Y | N | Y | Y | Y | Y | Y | Y | P | Y | Y | Y |
| [Gang Tang](https://pubmed.ncbi.nlm.nih.gov/?sort=pubdate&size=50&term=Tang+G&cauthor_id=33728708)，2021(Tang et al., 2021) | Y | P | Y | Y | N | Y | Y | Y | N | Y | Y | Y | Y | Y | Y | Y | Y | Y | Y | Y | Y | Y | Y | P | Y | Y | N |
| [M Ardiana](https://pubmed.ncbi.nlm.nih.gov/?sort=pubdate&size=50&term=Ardiana+M&cauthor_id=32454800)，2020(Ardiana et al., 2020) | Y | P | Y | Y | N | Y | Y | N | N | Y | Y | Y | Y | Y | Y | N | N | Y | Y | Y | Y | N | N | P | Y | N | N |
| [Jamal Hallajzadeh](https://pubmed.ncbi.nlm.nih.gov/?sort=pubdate&size=50&term=Hallajzadeh+J&cauthor_id=32394508),2020(Hallajzadeh et al., 2020) | Y | Y | Y | Y | N | Y | Y | N | Y | Y | Y | Y | Y | Y | Y | N | N | P | Y | Y | Y | Y | Y | P | Y | P | Y |
| [Mohsen Mohit](https://pubmed.ncbi.nlm.nih.gov/?sort=pubdate&size=50&term=Mohit+M&cauthor_id=33183658)，2020(Mohit et al., 2020) | Y | Y | Y | Y | N | Y | Y | N | Y | Y | Y | Y | Y | Y | Y | Y | Y | P | Y | Y | Y | Y | Y | P | Y | Y | Y |
| [Elham Razmpoosh](https://pubmed.ncbi.nlm.nih.gov/?sort=pubdate&size=50&term=Razmpoosh+E&cauthor_id=32201245)，2020(Razmpoosh et al., 2020) | Y | Y | Y | Y | Y | Y | Y | Y | Y | Y | Y | Y | Y | Y | Y | Y | Y | Y | Y | Y | Y | Y | Y | P | Y | Y | Y |
| [Rahele Tavakoly](https://pubmed.ncbi.nlm.nih.gov/?sort=pubdate&size=50&term=Tavakoly+R&cauthor_id=31331553)，2019(Tavakoly et al., 2019) | Y | Y | Y | Y | N | Y | Y | N | Y | Y | Y | Y | Y | Y | Y | Y | N | Y | Y | Y | Y | Y | Y | P | Y | Y | N |
| [Seyed Mohammad Mousavi](https://pubmed.ncbi.nlm.nih.gov/?sort=pubdate&size=50&term=Mousavi+SM&cauthor_id=29857879)，2018(Mousavi et al., 2018) | Y | P | Y | Y | N | Y | Y | Y | N | Y | Y | Y | Y | Y | Y | Y | N | Y | Y | Y | Y | Y | Y | P | Y | Y | N |
| [Nazli Namazi](https://pubmed.ncbi.nlm.nih.gov/?sort=pubdate&size=50&term=Namazi+N&cauthor_id=29559374)，2018(Namazi et al., 2018) | Y | Y | Y | Y | N | Y | Y | N | P | Y | Y | Y | Y | Y | Y | Y | N | Y | Y | Y | Y | Y | Y | P | Y | P | Y |
| [Reza Daryabeygi-Khotbehsara](https://pubmed.ncbi.nlm.nih.gov/?sort=pubdate&size=50&term=Daryabeygi-Khotbehsara+R&cauthor_id=29154069)，2017(Daryabeygi-Khotbehsara et al., 2017) | Y | P | Y | Y | N | Y | Y | P | N | Y | Y | Y | Y | Y | Y | Y | N | Y | Y | Y | Y | Y | Y | P | Y | Y | Y |
| [Amirhossein Sahebkar](https://pubmed.ncbi.nlm.nih.gov/?sort=pubdate&size=50&term=Sahebkar+A&cauthor_id=26875640)，2016(Sahebkar et al., 2016a) | Y | Y | Y | Y | N | Y | Y | N | N | N | Y | Y | Y | Y | Y | Y | N | Y | Y | Y | Y | Y | Y | P | Y | Y | Y |
| [Amirhossein Sahebkar](https://pubmed.ncbi.nlm.nih.gov/?sort=pubdate&size=50&term=Sahebkar+A&cauthor_id=27512971) ，2016(Sahebkar et al., 2016b) | Y | P | Y | Y | N | Y | Y | N | N | Y | Y | Y | Y | Y | Y | Y | N | Y | Y | Y | Y | Y | Y | P | Y | Y | N |

Notes: Item 1: Title; Item2: Structured summary; Item 3: Rationale; Item 4: Objectives; Item 5: Protocol and registration; Item 6: Eligibility criteria; Item 7: Information sources; Item 8: Search; Item 9: Study selection; Item 10：Data collection process; Item 11: Data items; Item 12: Risk of bias in individual studies; Item 13: Summary measures; Item 14: Synthesis of results; Item 15: Risk of bias across studies; Item 16: Additional analyses; Item 17: Study selection; Item 18: Study characteristics; Item 19: Risk of bias within studies; Item 20: Results of individual studies; Item 21: Synthesis of results; Item 22: Risk of bias across studies; Item 23: Additional analysis; Item 24: Summary of evidence; Item 25: Limitations; Item 26: Conclusions; Item 27: Funding.

References：

Ardiana, M., Pikir, B.S., Santoso, A., Hermawan, H.O., and Al-Farabi, M.J. (2020). Effect of Supplementation on Oxidative Stress and Antioxidant Parameters: A Meta-Analysis of Randomized Controlled Trials. *TheScientificWorldJournal* 2020**,** 2390706. doi: 10.1155/2020/2390706.

Azizi, N., Amini, M.R., Djafarian, K., and Shab-Bidar, S. (2021). The Effects of Supplementation on Liver Enzymes Levels: a Systematic Review and Meta-analysis of Randomized Controlled Trials. *Clinical Nutrition Research* 10(1)**,** 72-82. doi: 10.7762/cnr.2021.10.1.72.

Chattopadhyay, K., Wang, H., Kaur, J., Nalbant, G., Almaqhawi, A., Kundakci, B., et al. (2022). Effectiveness and Safety of Ayurvedic Medicines in Type 2 Diabetes Mellitus Management: A Systematic Review and Meta-Analysis. *Frontiers In Pharmacology* 13**,** 821810. doi: 10.3389/fphar.2022.821810.

Daryabeygi-Khotbehsara, R., Golzarand, M., Ghaffari, M.P., and Djafarian, K. (2017). Nigella sativa improves glucose homeostasis and serum lipids in type 2 diabetes: A systematic review and meta-analysis. *Complementary Therapies In Medicine* 35. doi: 10.1016/j.ctim.2017.08.016.

Golpour-Hamedani, S., Hadi, A., SafariMalekabadi, D., Najafgholizadeh, A., Askari, G., and Pourmasoumi, M. (2022). The effect of nigella supplementation on blood pressure: A systematic review and dose-response meta-analysis. *Critical Reviews In Food Science and Nutrition*. doi: 10.1080/10408398.2022.2110566.

Gyawali, D., Vohra, R., Orme-Johnson, D., Ramaratnam, S., and Schneider, R.H. (2021). A Systematic Review and Meta-Analysis of Ayurvedic Herbal Preparations for Hypercholesterolemia. *Medicina (Kaunas, Lithuania)* 57(6)**,** 546. doi: 10.3390/medicina57060546.

Hallajzadeh, J., Milajerdi, A., Mobini, M., Amirani, E., Azizi, S., Nikkhah, E., et al. (2020). Effects of Nigella sativa on glycemic control, lipid profiles, and biomarkers of inflammatory and oxidative stress: A systematic review and meta-analysis of randomized controlled clinical trials. *Phytotherapy Research : PTR* 34(10)**,** 2586-2608. doi: 10.1002/ptr.6708.

Han, A., and Shi, D. (2021). The efficacy of Nigella sativa supplementation for asthma control: a meta-analysis of randomized controlled studies. *Postepy Dermatologii I Alergologii* 38(4)**,** 561-565. doi: 10.5114/ada.2020.93220.

Malekian, S., Ghassab-Abdollahi, N., Mirghafourvand, M., and Farshbaf-Khalili, A. (2021). The effect of Nigella Sativa on oxidative stress and inflammatory biomarkers: a systematic review and meta-analysis. *Journal of Complementary & Integrative Medicine* 18(2)**,** 235-259. doi: 10.1515/jcim-2019-0198.

Mohit, M., Farrokhzad, A., Faraji, S.N., Heidarzadeh-Esfahani, N., and Kafeshani, M. (2020). Effect of Nigella sativa L. supplementation on inflammatory and oxidative stress indicators: A systematic review and meta-analysis of controlled clinical trials. *Complementary Therapies In Medicine* 54**,** 102535. doi: 10.1016/j.ctim.2020.102535.

Montazeri, R.S., Fatahi, S., Sohouli, M.H., Abu-Zaid, A., Santos, H.O., Găman, M.-A., et al. (2021). The effect of nigella sativa on biomarkers of inflammation and oxidative stress: A systematic review and meta-analysis of randomized controlled trials. *Journal of Food Biochemistry* 45(4)**,** e13625. doi: 10.1111/jfbc.13625.

Mousavi, S.M., Sheikhi, A., Varkaneh, H.K., Zarezadeh, M., Rahmani, J., and Milajerdi, A. (2018). Effect of Nigella sativa supplementation on obesity indices: A systematic review and meta-analysis of randomized controlled trials. *Complementary Therapies In Medicine* 38**,** 48-57. doi: 10.1016/j.ctim.2018.04.003.

Namazi, N., Larijani, B., Ayati, M.H., and Abdollahi, M. (2018). The effects of Nigella sativa L. on obesity: A systematic review and meta-analysis. *Journal of Ethnopharmacology* 219**,** 173-181. doi: 10.1016/j.jep.2018.03.001.

Razmpoosh, E., Safi, S., Abdollahi, N., Nadjarzadeh, A., Nazari, M., Fallahzadeh, H., et al. (2020). The effect of Nigella sativa on the measures of liver and kidney parameters: A systematic review and meta-analysis of randomized-controlled trials. *Pharmacological Research* 156**,** 104767. doi: 10.1016/j.phrs.2020.104767.

Saadati, S., Naseri, K., Asbaghi, O., Abhari, K., Zhang, P., Li, H.-B., et al. (2022). Nigella sativa supplementation improves cardiometabolic indicators in population with prediabetes and type 2 diabetes mellitus: A systematic review and meta-analysis of randomized controlled trials. *Frontiers In Nutrition* 9**,** 977756. doi: 10.3389/fnut.2022.977756.

Sahebkar, A., Beccuti, G., Simental-Mendía, L.E., Nobili, V., and Bo, S. (2016a). Nigella sativa (black seed) effects on plasma lipid concentrations in humans: A systematic review and meta-analysis of randomized placebo-controlled trials. *Pharmacological Research* 106**,** 37-50. doi: 10.1016/j.phrs.2016.02.008.

Sahebkar, A., Soranna, D., Liu, X., Thomopoulos, C., Simental-Mendia, L.E., Derosa, G., et al. (2016b). A systematic review and meta-analysis of randomized controlled trials investigating the effects of supplementation with Nigella sativa (black seed) on blood pressure. *Journal of Hypertension* 34(11)**,** 2127-2135. doi: 10.1097/HJH.0000000000001049.

Tang, G., Zhang, L., Tao, J., and Wei, Z. (2021). Effect of Nigella sativa in the treatment of nonalcoholic fatty liver disease: A systematic review and meta-analysis of randomized controlled trials. *Phytotherapy Research : PTR* 35(8)**,** 4183-4193. doi: 10.1002/ptr.7080.

Tavakoly, R., Arab, A., Vallianou, N., Clark, C.C.T., Hadi, A., Ghaedi, E., et al. (2019). The effect of Nigella sativa L. supplementation on serum C-reactive protein: A systematic review and meta-analysis of randomized controlled trials. *Complementary Therapies In Medicine* 45**,** 149-155. doi: 10.1016/j.ctim.2019.06.008.

Tiwari, A., G, S., Meka, S., Varghese, B., Vishwakarma, G., and Adela, R. (2022). The effect of Nigella sativa on non-alcoholic fatty liver disease: A systematic review and meta-analysis. *Human Nutrition and Metabolism* 28. doi: 10.1016/j.hnm.2022.200146.
