# Supplementary material for: Nigella sativa and health outcomes: An overview of systematic reviews and meta-analyses
Source: Front Nutr. 2023 Mar 28;10:1107750. doi: 10.3389/fnut.2023.1107750 (PMC10086143; doi:10.3389/fnut.2023.1107750)
Supplement: Supplementary file 3 [file Table_3.DOCX]

**Supplementary Table S3 Results of methodological quality**

| **Reference** | **Item 1** | **Item 2** | **Item 3** | **Item 4** | **Item 5** | **Item 6** | **Item 7** | **Item 8** | **Item 9** | **Item 10** | **Item 11** | **Item 12** | **Item 13** | **Item 14** | **Item 15** | **Item 16** | **Overall quality** |
| --- | --- | --- | --- | --- | --- | --- | --- | --- | --- | --- | --- | --- | --- | --- | --- | --- | --- |
| [Saeede Saadati](https://pubmed.ncbi.nlm.nih.gov/?size=50&term=Saadati+S&cauthor_id=36034891)，2022 (Saadati et al., 2022) | Y | N | N | P | Y | Y | N | Y | Y | N | Y | Y | N | Y | Y | Y | **Critically low** |
| Anoop Tiwari，2022 (Tiwari et al., 2022) | Y | Y | N | P | Y | N | N | Y | Y | N | Y | Y | Y | Y | Y | Y | **Low** |
| Sahar Golpour-hamedani, 2022 (Golpour-Hamedani et al., 2022) | Y | Y | N | P | Y | Y | N | Y | Y | N | Y | Y | Y | Y | Y | Y | **Low** |
| Kaushik Chattopadhyay, 2022 (Chattopadhyay et al., 2022) | Y | Y | N | P | Y | Y | Y | Y | P | Y | Y | N | N | Y | N | Y | **Critically low** |
| [Neda Azizi](https://pubmed.ncbi.nlm.nih.gov/?sort=pubdate&size=50&term=Azizi+N&cauthor_id=33564654)，2021 (Azizi et al., 2021) | Y | N | N | P | N | Y | N | Y | Y | N | Y | Y | Y | N | Y | Y | **Critically low** |
| [Dinesh Gyawali](https://pubmed.ncbi.nlm.nih.gov/?sort=pubdate&size=50&term=Gyawali+D&cauthor_id=34071454)，2021 (Gyawali et al., 2021) | Y | Y | N | Y | Y | N | N | Y | Y | N | Y | N | N | N | N | Y | **Critically low** |
| [Anqiang Han](https://pubmed.ncbi.nlm.nih.gov/?sort=pubdate&size=50&term=Han+A&cauthor_id=34658694)，2021 (Han and Shi, 2021) | Y | N | N | P | N | Y | N | P | P | N | Y | N | N | Y | N | Y | **Critically low** |
| [Sanaz Malekian](https://pubmed.ncbi.nlm.nih.gov/?sort=pubdate&size=50&term=Malekian+S&cauthor_id=34187123)，2021 (Malekian et al., 2021) | Y | N | N | P | Y | Y | N | Y | Y | N | Y | N | N | Y | N | Y | **Critically low** |
| [Rahele Sadat Montazeri](https://pubmed.ncbi.nlm.nih.gov/?sort=pubdate&size=50&term=Montazeri+RS&cauthor_id=33559935)，2021 (Montazeri et al., 2021) | Y | N | N | P | N | Y | N | Y | P | N | Y | Y | Y | Y | Y | Y | **Critically low** |
| [Gang Tang](https://pubmed.ncbi.nlm.nih.gov/?sort=pubdate&size=50&term=Tang+G&cauthor_id=33728708)，2021 (Tang et al., 2021) | Y | N | N | P | Y | Y | Y | Y | Y | N | Y | Y | Y | Y | Y | Y | **Low** |
| [M Ardiana](https://pubmed.ncbi.nlm.nih.gov/?sort=pubdate&size=50&term=Ardiana+M&cauthor_id=32454800)，2020 (Ardiana et al., 2020) | Y | N | N | P | N | Y | N | Y | P | N | Y | N | N | Y | N | Y | **Critically low** |
| [Jamal Hallajzadeh](https://pubmed.ncbi.nlm.nih.gov/?sort=pubdate&size=50&term=Hallajzadeh+J&cauthor_id=32394508),2020 (Hallajzadeh et al., 2020) | Y | N | N | P | Y | Y | N | P | Y | N | Y | Y | Y | Y | Y | Y | **Critically low** |
| [Mohsen Mohit](https://pubmed.ncbi.nlm.nih.gov/?sort=pubdate&size=50&term=Mohit+M&cauthor_id=33183658)，2020 (Mohit et al., 2020) | Y | N | N | P | Y | Y | Y | P | P | N | Y | Y | Y | Y | Y | Y | **Low** |
| [Elham Razmpoosh](https://pubmed.ncbi.nlm.nih.gov/?sort=pubdate&size=50&term=Razmpoosh+E&cauthor_id=32201245)，2020 (Razmpoosh et al., 2020) | Y | Y | N | P | Y | Y | Y | Y | Y | N | Y | Y | Y | Y | Y | Y | **Moderate** |
| [Rahele Tavakoly](https://pubmed.ncbi.nlm.nih.gov/?sort=pubdate&size=50&term=Tavakoly+R&cauthor_id=31331553)，2019 (Tavakoly et al., 2019) | Y | N | N | P | Y | Y | N | Y | P | N | Y | Y | Y | Y | Y | N | **Critically low** |
| [Seyed Mohammad Mousavi](https://pubmed.ncbi.nlm.nih.gov/?sort=pubdate&size=50&term=Mousavi+SM&cauthor_id=29857879)，2018 (Mousavi et al., 2018) | Y | N | N | P | N | Y | N | Y | P | N | Y | Y | Y | Y | Y | Y | **Critically low** |
| [Nazli Namazi](https://pubmed.ncbi.nlm.nih.gov/?sort=pubdate&size=50&term=Namazi+N&cauthor_id=29559374)，2018 (Namazi et al., 2018) | Y | N | N | P | Y | Y | N | Y | P | N | Y | Y | Y | Y | Y | Y | **Critically low** |
| [Reza Daryabeygi-Khotbehsara](https://pubmed.ncbi.nlm.nih.gov/?sort=pubdate&size=50&term=Daryabeygi-Khotbehsara+R&cauthor_id=29154069)，2017 (Daryabeygi-Khotbehsara et al., 2017) | Y | N | N | P | Y | Y | N | Y | P | N | Y | Y | Y | Y | Y | N | **Critically low** |
| [Amirhossein Sahebkar](https://pubmed.ncbi.nlm.nih.gov/?sort=pubdate&size=50&term=Sahebkar+A&cauthor_id=26875640)，2016 (Sahebkar et al., 2016a) | Y | N | N | P | N | N | N | Y | Y | N | Y | Y | Y | Y | Y | Y | **Critically low** |
| [Amirhossein Sahebkar](https://pubmed.ncbi.nlm.nih.gov/?sort=pubdate&size=50&term=Sahebkar+A&cauthor_id=27512971) ，2016 (Sahebkar et al., 2016b) | Y | N | N | P | N | Y | N | Y | Y | N | Y | Y | Y | Y | Y | Y | **Critically low** |

Abbreviations: Y: Yes; N: No; P: Partial yes.

Item 1: Did the research questions and inclusion criteria for the review include the components of PICO?; Item 2: Did the report of the review contain an explicit statement that the review methods were established prior to the conduct of the review and did the report justify any significant deviations from the protocol?; Item 3: Did the review authors explain their selection of the study designs for inclusion in the review?; Item 4: Did the review authors use a comprehensive literature search strategy?; Item 5: Did the review authors perform study selection in duplicate?; Item 6: Did the review authors perform data extraction in duplicate?; Item 7: Did the review authors provide a list of excluded studies and justify the exclusions?; Item 8: Did the review authors describe the included studies in adequate detail?; Item 9: Did the review authors use a satisfactory technique for assessing the risk of bias (RoB) in individual studies that were included in the review?; Item 10: Did the review authors report on the sources of funding for the studies included in the review?; Item 11: If meta-analysis was performed did the review authors use appropriate methods for statistical combination of results?; Item 12: If meta-analysis was performed, did the review authors assess the potential impact of RoB in individual studies on the results of the meta-analysis or other evidence synthesis?; Item 13: Did the review authors account for RoB in individual studies when interpreting/discussing the results of the review?; Item 14: Did the review authors provide a satisfactory explanation for, and discussion of, any heterogeneity observed in the results of the review?; Item 15: If they performed quantitative synthesis did the review authors carry out an adequate investigation of publication bias (small study bias) and discuss its likely impact on the results of the review?; Item 16: Did the review authors report any potential sources of conflict of interest, including any funding they received for conducting the review?.

Reeferences：

Ardiana, M., Pikir, B.S., Santoso, A., Hermawan, H.O., and Al-Farabi, M.J. (2020). Effect of Supplementation on Oxidative Stress and Antioxidant Parameters: A Meta-Analysis of Randomized Controlled Trials. *TheScientificWorldJournal* 2020**,** 2390706. doi: 10.1155/2020/2390706.

Azizi, N., Amini, M.R., Djafarian, K., and Shab-Bidar, S. (2021). The Effects of Supplementation on Liver Enzymes Levels: a Systematic Review and Meta-analysis of Randomized Controlled Trials. *Clinical Nutrition Research* 10(1)**,** 72-82. doi: 10.7762/cnr.2021.10.1.72.

Chattopadhyay, K., Wang, H., Kaur, J., Nalbant, G., Almaqhawi, A., Kundakci, B., et al. (2022). Effectiveness and Safety of Ayurvedic Medicines in Type 2 Diabetes Mellitus Management: A Systematic Review and Meta-Analysis. *Frontiers In Pharmacology* 13**,** 821810. doi: 10.3389/fphar.2022.821810.

Daryabeygi-Khotbehsara, R., Golzarand, M., Ghaffari, M.P., and Djafarian, K. (2017). Nigella sativa improves glucose homeostasis and serum lipids in type 2 diabetes: A systematic review and meta-analysis. *Complementary Therapies In Medicine* 35. doi: 10.1016/j.ctim.2017.08.016.

Golpour-Hamedani, S., Hadi, A., SafariMalekabadi, D., Najafgholizadeh, A., Askari, G., and Pourmasoumi, M. (2022). The effect of nigella supplementation on blood pressure: A systematic review and dose-response meta-analysis. *Critical Reviews In Food Science and Nutrition*. doi: 10.1080/10408398.2022.2110566.

Gyawali, D., Vohra, R., Orme-Johnson, D., Ramaratnam, S., and Schneider, R.H. (2021). A Systematic Review and Meta-Analysis of Ayurvedic Herbal Preparations for Hypercholesterolemia. *Medicina (Kaunas, Lithuania)* 57(6)**,** 546. doi: 10.3390/medicina57060546.

Hallajzadeh, J., Milajerdi, A., Mobini, M., Amirani, E., Azizi, S., Nikkhah, E., et al. (2020). Effects of Nigella sativa on glycemic control, lipid profiles, and biomarkers of inflammatory and oxidative stress: A systematic review and meta-analysis of randomized controlled clinical trials. *Phytotherapy Research : PTR* 34(10)**,** 2586-2608. doi: 10.1002/ptr.6708.

Han, A., and Shi, D. (2021). The efficacy of Nigella sativa supplementation for asthma control: a meta-analysis of randomized controlled studies. *Postepy Dermatologii I Alergologii* 38(4)**,** 561-565. doi: 10.5114/ada.2020.93220.

Malekian, S., Ghassab-Abdollahi, N., Mirghafourvand, M., and Farshbaf-Khalili, A. (2021). The effect of Nigella Sativa on oxidative stress and inflammatory biomarkers: a systematic review and meta-analysis. *Journal of Complementary & Integrative Medicine* 18(2)**,** 235-259. doi: 10.1515/jcim-2019-0198.

Mohit, M., Farrokhzad, A., Faraji, S.N., Heidarzadeh-Esfahani, N., and Kafeshani, M. (2020). Effect of Nigella sativa L. supplementation on inflammatory and oxidative stress indicators: A systematic review and meta-analysis of controlled clinical trials. *Complementary Therapies In Medicine* 54**,** 102535. doi: 10.1016/j.ctim.2020.102535.

Montazeri, R.S., Fatahi, S., Sohouli, M.H., Abu-Zaid, A., Santos, H.O., Găman, M.-A., et al. (2021). The effect of nigella sativa on biomarkers of inflammation and oxidative stress: A systematic review and meta-analysis of randomized controlled trials. *Journal of Food Biochemistry* 45(4)**,** e13625. doi: 10.1111/jfbc.13625.

Mousavi, S.M., Sheikhi, A., Varkaneh, H.K., Zarezadeh, M., Rahmani, J., and Milajerdi, A. (2018). Effect of Nigella sativa supplementation on obesity indices: A systematic review and meta-analysis of randomized controlled trials. *Complementary Therapies In Medicine* 38**,** 48-57. doi: 10.1016/j.ctim.2018.04.003.

Namazi, N., Larijani, B., Ayati, M.H., and Abdollahi, M. (2018). The effects of Nigella sativa L. on obesity: A systematic review and meta-analysis. *Journal of Ethnopharmacology* 219**,** 173-181. doi: 10.1016/j.jep.2018.03.001.

Razmpoosh, E., Safi, S., Abdollahi, N., Nadjarzadeh, A., Nazari, M., Fallahzadeh, H., et al. (2020). The effect of Nigella sativa on the measures of liver and kidney parameters: A systematic review and meta-analysis of randomized-controlled trials. *Pharmacological Research* 156**,** 104767. doi: 10.1016/j.phrs.2020.104767.

Saadati, S., Naseri, K., Asbaghi, O., Abhari, K., Zhang, P., Li, H.-B., et al. (2022). Nigella sativa supplementation improves cardiometabolic indicators in population with prediabetes and type 2 diabetes mellitus: A systematic review and meta-analysis of randomized controlled trials. *Frontiers In Nutrition* 9**,** 977756. doi: 10.3389/fnut.2022.977756.

Sahebkar, A., Beccuti, G., Simental-Mendía, L.E., Nobili, V., and Bo, S. (2016a). Nigella sativa (black seed) effects on plasma lipid concentrations in humans: A systematic review and meta-analysis of randomized placebo-controlled trials. *Pharmacological Research* 106**,** 37-50. doi: 10.1016/j.phrs.2016.02.008.

Sahebkar, A., Soranna, D., Liu, X., Thomopoulos, C., Simental-Mendia, L.E., Derosa, G., et al. (2016b). A systematic review and meta-analysis of randomized controlled trials investigating the effects of supplementation with Nigella sativa (black seed) on blood pressure. *Journal of Hypertension* 34(11)**,** 2127-2135. doi: 10.1097/HJH.0000000000001049.

Tang, G., Zhang, L., Tao, J., and Wei, Z. (2021). Effect of Nigella sativa in the treatment of nonalcoholic fatty liver disease: A systematic review and meta-analysis of randomized controlled trials. *Phytotherapy Research : PTR* 35(8)**,** 4183-4193. doi: 10.1002/ptr.7080.

Tavakoly, R., Arab, A., Vallianou, N., Clark, C.C.T., Hadi, A., Ghaedi, E., et al. (2019). The effect of Nigella sativa L. supplementation on serum C-reactive protein: A systematic review and meta-analysis of randomized controlled trials. *Complementary Therapies In Medicine* 45**,** 149-155. doi: 10.1016/j.ctim.2019.06.008.

Tiwari, A., G, S., Meka, S., Varghese, B., Vishwakarma, G., and Adela, R. (2022). The effect of Nigella sativa on non-alcoholic fatty liver disease: A systematic review and meta-analysis. *Human Nutrition and Metabolism* 28. doi: 10.1016/j.hnm.2022.200146.
