# Supplementary material for: Nigella sativa and health outcomes: An overview of systematic reviews and meta-analyses
Source: Front Nutr. 2023 Mar 28;10:1107750. doi: 10.3389/fnut.2023.1107750 (PMC10086143; doi:10.3389/fnut.2023.1107750)
Supplement: Supplementary file 4 [file Table_4.DOCX]

Supplementary Material

**Supplementary Table S4 GRADE profile of *N. sativa* for health outcomes**

| **Reference** | **Outcomes** | **Population** | **Interventions/ comparators** | **Number of RCTs** | **Sample size** | **Effect metrics** | **Estimates** | **95%CI** | **I^2^** | **P value** | **Risk of bias** | **Inconsistency** | **Indirection** | **Imprecision** | **publication bias** | **GRADE level** |
| --- | --- | --- | --- | --- | --- | --- | --- | --- | --- | --- | --- | --- | --- | --- | --- | --- |
| [Saeede Saadati](https://pubmed.ncbi.nlm.nih.gov/?size=50&term=Saadati+S&cauthor_id=36034891)，2022(Saadati et al., 2022) | BMI | Prediabetes and T2DM | *N. sativa/*Placebo and routine therapies | 5 | 275 | WMD | -0.56 | -1.95, 0.81 | 83.2% | <0.001 | Serious ^a^ | Very serious ^b^ | Not serious | Very serious ^c, d^ | Not serious | **⨁◯◯◯**  **Very low** |
|  | FPG | Prediabetes and T2DM | *N. sativa/*Placebo, and routine therapies | 10 | 623 | WMD | -24.18 | -39.36, -9 | 98.7% | 0.002 | Serious ^a^ | Very serious ^b^ | Not serious | Not serious | Not serious | **⨁◯◯◯**  **Very low** |
|  | HbA1c | Prediabetes and T2DM | *N. sativa/*Placebo and routine therapies | 7 | 490 | WMD | -0.54 | -0.82, -0.26 | 94.4% | <0.001 | Serious ^a^ | Very serious ^b^ | Not serious | Not serious | Serious ^f^ | **⨁◯◯◯**  **Very low** |
|  | OGTT | Prediabetes and T2DM | *N. sativa/*Placebo and routine therapies | 4 | 283 | WMD | -12.28 | -29.94, 5.38 | 90.7% | 0.173 | Serious ^a^ | Very serious ^b^ | Not serious | Very serious ^c, d^ | Serious ^f^ | **⨁◯◯◯**  **Very low** |
|  | Fasting insulin | Prediabetes and T2DM | *N. sativa/*Placebo and routine therapies | 5 | 246 | WMD | 1.06 | -1.87,4 | 95.8% | <0.001 | Serious ^a^ | Very serious ^b^ | Not serious | Very serious ^c, d^ | Not serious | **⨁◯◯◯**  **Very low** |
|  | HOMA-IR | Prediabetes and T2DM | *N. sativa/*Placebo and routine therapies | 5 | 319 | WMD | -0.20 | -0.92, 0.50 | 88.6% | 0.572 | Serious ^a^ | Very serious ^b^ | Not serious | Serious ^d^ | Not serious | **⨁◯◯◯**  **Very low** |
|  | TG | Prediabetes and T2DM | *N. sativa/*Placebo and routine therapies | 7 | 405 | WMD | -11.85 | -29.83, 6.12 | 90.1% | 0.196 | Serious ^a^ | Very serious ^b^ | Not serious | Serious ^d^ | Not serious | **⨁◯◯◯**  **Very low** |
|  | TC | Prediabetes and T2DM | *N. sativa/*Placebo and routine therapies | 6 | 325 | WMD | -23.84 | -39.25, -8.44 | 91.9% | 0.002 | Serious ^a^ | Very serious ^b^ | Not serious | Not serious | Serious ^f^ | **⨁◯◯◯**  **Very low** |
|  | LDL-C | Prediabetes and T2DM | *N. sativa/*Placebo and routine therapies | 7 | 325 | WMD | -20.12 | -33.72, -6.51 | 92.6% | 0.004 | Serious ^a^ | Very serious ^b^ | Not serious | Not serious | Not serious | **⨁◯◯◯**  **Very low** |
|  | HDL-C | Prediabetes and T2DM | *N. sativa/*Placebo and routine therapies | 7 | 325 | WMD | 0.56 | -1.98, 3.11 | 87.3% | 0.663 | Serious ^a^ | Very serious ^b^ | Not serious | Serious ^d^ | Not serious | **⨁◯◯◯**  **Very low** |
|  | CRP | Prediabetes and T2DM | *N. sativa/*Placebo and routine therapies | 3 | 111 | WMD | -1.05 | -1.75, -0.35 | 85.4% | 0.003 | Serious ^a^ | Very serious ^b^ | Not serious | Serious ^c^ | Not serious | **⨁◯◯◯**  **Very low** |
|  | MDA | Prediabetes and T2DM | *N. sativa/*Placebo and routine therapies | 3 | 274 | WMD | -1.27 | -2.53, -0.01 | 90.3% | 0.048 | Serious ^a^ | Very serious ^b^ | Not serious | Serious ^c^ | Not serious | **⨁◯◯◯**  **Very low** |
| Anoop Tiwari，2022(Tiwari et al., 2022) | ALT | NAFLD | *N. sativa/*Placebo | 4 | 284 | SMD | 2.09 | 0.57, 3.61 | 96% | 0.007 | Serious ^a^ | Very serious ^b^ | Not serious | Serious ^c^ | Not serious | **⨁◯◯◯**  **Very low** |
|  | AST | NAFLD | *N. sativa/*Placebo | 4 | 284 | SMD | 1.85 | 0.50, 3.19 | 95% | 0.007 | Serious ^a^ | Very serious ^b^ | Not serious | Serious ^c^ | Not serious | **⨁◯◯◯**  **Very low** |
|  | BMI | NAFLD | *N. sativa/*Placebo | 4 | 284 | SMD | 0.16 | -0.08, 0.40 | 2% | 0.18 | Serious ^a^ | Not serious | Not serious | Very serious ^c, d^ | Not serious | **⨁◯◯◯**  **Very low** |
|  | LDL-C | NAFLD | *N. sativa/*Placebo | 4 | 284 | SMD | 0.55 | 0.06, 1.03 | 63% | 0.03 | Serious ^a^ | Serious ^e^ | Not serious | Serious ^c^ | Not serious | **⨁◯◯◯**  **Very low** |
|  | HDL-C | NAFLD | *N. sativa/*Placebo | 4 | 284 | SMD | 0.82 | 1.52, 0.12 | 80% | 0.02 | Serious ^a^ | Very serious ^b^ | Not serious | Serious ^c^ | Not serious | **⨁◯◯◯**  **Very low** |
|  | TG | NAFLD | *N. sativa/*Placebo | 4 | 284 | SMD | 0.37 | -0.10, 0.64 | 0% | 0.008 | Serious ^a^ | Not serious | Not serious | Very serious ^c, d^ | Not serious | **⨁◯◯◯**  **Very low** |
| Sahar Golpour-hamedani, 2022 (Golpour-Hamedani et al., 2022) | SBP | Adults | *N. sativa/*Placebo, standard therapy | 17 | 1048 | MD | -4.58 | -6.22, -2.94 | 81% | <0.000001 | Serious ^a^ | Very serious ^b^ | Not serious | Not serious | Serious ^f^ | **⨁◯◯◯**  **Very low** |
|  | DBP | Adults | *N. sativa/*Placebo, standard therapy | 17 | 1048 | MD | -3.08 | -4.62, -1.55 | 82% | <0.000001 | Serious ^a^ | Very serious ^b^ | Not serious | Not serious | Not serious | **⨁◯◯◯**  **Very low** |
| Kaushik Chattopadhyay, 2022(Chattopadhyay et al., 2022) | FBG | T2DM | *N. sativa/*Placebo and no treatment | 7 | 458 | MD | -14.77 | -29.92, 0.37 | 86.51% | 0.06 | Serious ^a^ | Very serious ^b^ | Not serious | Serious ^d^ | Not mention | **⨁◯◯◯**  **Very low** |
|  | HbA1c | T2DM | *N. sativa/* Placebo and no treatment | 4 | 324 | MD | -0.36 | -0.64, -0.09 | 53.94% | 0.01 | Serious ^a^ | Serious ^e^ | Not serious | Not serious | Not mention | **⨁⨁◯◯**  **Low** |
|  | HOMA-IR | T2DM | *N. sativa/*Placebo | 3 | 215 | MD | -0.61 | -1.49, 0.26 | 83.75% | 0.17 | Serious ^a^ | Very serious ^b^ | Not serious | Very serious ^c, d^ | Not mention | **⨁◯◯◯**  **Very low** |
|  | Fasting insulin | T2DM | *N. sativa/*Placebo | 2 | 112 | MD | -1.78 | -3.55, -0.00 | 47.83% | 0.05 | Serious ^a^ | Not serious | Not serious | Very serious ^c, d^ | Not mention | **⨁◯◯◯**  **Very low** |
|  | PPBG | T2DM | *N. sativa/* Placebo and no treatment | 2 | 150 | MD | -10.26 | -35.23, 14.71 | 82.26% | 0.42 | Serious ^a^ | Very serious ^b^ | Not serious | Very serious ^c, d^ | Not mention | **⨁◯◯◯**  **Very low** |
|  | BW | T2DM | *N. sativa/*Placebo | 2 | 112 | MD | -4.20 | -7.21, -1.19 | 0% | 0.01 | Serious ^a^ | Not serious | Not serious | Very serious ^c, d^ | Not mention | **⨁◯◯◯**  **Very low** |
|  | BMI | T2DM | *N. sativa/*Placebo | 3 | 182 | MD | -0.87 | -1.94, 0.20 | 0% | 0.11 | Serious ^a^ | Not serious | Not serious | Very serious ^c, d^ | Not mention | **⨁◯◯◯**  **Very low** |
|  | HDL-C | T2DM | *N. sativa/* Placebo and no treatment | 6 | 355 | MD | 2.83 | -0.78, 6.43 | 79.97% | 0.12 | Serious ^a^ | Very serious ^b^ | Not serious | Serious ^d^ | Not mention | **⨁◯◯◯**  **Very low** |
|  | LDL-C | T2DM | *N. sativa/* Placebo and no treatment | 6 | 355 | MD | -11.25 | -16.53, -5.98 | 0% | 0.00 | Serious ^a^ | Not serious | Not serious | Not serious | Not mention | **⨁⨁⨁◯**  **Moderate** |
|  | TC | T2DM | *N. sativa/* Placebo and no treatment | 5 | 274 | MD | -16.84 | -30.70, -2.97 | 59.78% | 0.02 | Serious ^a^ | Serious ^e^ | Not serious | Serious ^c^ | Not mention | **⨁◯◯◯**  **Very low** |
|  | TG | T2DM | *N. sativa/* Placebo and no treatment | 6 | 355 | MD | -12.16 | -21.02, -3.30 | 0.41% | 0.01 | Serious ^a^ | Not serious | Not serious | Not serious | Not mention | **⨁⨁⨁◯**  **Moderate** |
| [Neda Azizi](https://pubmed.ncbi.nlm.nih.gov/?sort=pubdate&size=50&term=Azizi+N&cauthor_id=33564654)，2021(Azizi et al., 2021) | ALT | Adults | *N. sativa/*Placebo | 8 | NR | WMD | -7.26 | -15.42, 0.04 | 97.8% | 0.001 | Serious ^a^ | Very serious ^b^ | Not serious | Serious ^d^ | Not mention | **⨁◯◯◯**  **Very low** |
|  | AST | Adults | *N. sativa/*Placebo | 7 | NR | WMD | -8.11 | -13.69, -2.53 | 95.9% | 0.004 | Serious ^a^ | Very serious ^b^ | Not serious | Not serious | Not mention | **⨁◯◯◯**  **Very low** |
| [Dinesh Gyawali](https://pubmed.ncbi.nlm.nih.gov/?sort=pubdate&size=50&term=Gyawali+D&cauthor_id=34071454)，2021(Gyawali et al., 2021) | TC | Hypercholesterolemia | *N. sativa/*Placebo | 3 | 163 | MD | -9.28 | -17.36, -1.19 | 0% | 0.02 | Serious ^a^ | Not serious | Not serious | Serious ^c^ | Not mention | **⨁⨁◯◯**  **Low** |
|  | LDL-C | Hypercholesterolemia | *N. sativa/*Placebo | 3 | 163 | MD | -2.12 | -7.85, 3.60 | 0% | 0.47 | Serious ^a^ | Not serious | Not serious | Very serious ^c, d^ | Not mention | **⨁◯◯◯**  **Very low** |
|  | TG | Hypercholesterolemia | *N. sativa/*Placebo | 3 | 163 | MD | -21.09 | -44.96, 2.77 | 28% | 0.08 | Serious ^a^ | Not serious | Not serious | Very serious ^c, d^ | Not mention | **⨁◯◯◯**  **Very low** |
|  | HDL-C | Hypercholesterolemia | *N. sativa/*Placebo | 3 | 163 | MD | 1.92 | -1.62, 5.45 | 21% | 0.29 | Serious ^a^ | Not serious | Not serious | Very serious ^c, d^ | Not mention | **⨁⨁◯◯**  **Low** |
| [Anqiang Han](https://pubmed.ncbi.nlm.nih.gov/?sort=pubdate&size=50&term=Han+A&cauthor_id=34658694)，2022(Han and Shi, 2021) | ACT | Asthma | *N. sativa/*Placebo | 2 | 108 | SMD | 0.50 | 0.11, 0.88 | 0% | 0.01 | Serious ^a^ | Not serious | Not serious | Serious ^c^ | Not mention | **⨁⨁◯◯**  **Low** |
|  | FEV _1_ | Asthma | *N. sativa/*Placebo | 3 | 128 | SMD | 1.84 | 0.07, 3.60 | 94% | 0.04 | Serious ^a^ | Very serious ^b^ | Not serious | Serious ^c^ | Not mention | **⨁◯◯◯**  **Very low** |
|  | PEF | Asthma | *N. sativa/*Placebo | 2 | 78 | SMD | 3.11 | -1.30, 7.52 | 96% | 0.17 | Serious ^a^ | Very serious ^b^ | Not serious | Very serious ^c, d^ | Not mention | **⨁◯◯◯**  **Very low** |
|  | IL-4 | Asthma | *N. sativa/*Placebo | 2 | 78 | SMD | -0.31 | -1.21, 0.59 | 72% | 0.5 | Serious ^a^ | Serious ^e^ | Not serious | Very serious ^c, d^ | Not mention | **⨁◯◯◯**  **Very low** |
|  | IFN-γ | Asthma | *N. sativa/*Placebo | 2 | 78 | SMD | 1.11 | -0.44, 2.67 | 88% | 0.16 | Serious ^a^ | Very serious ^b^ | Not serious | Very serious ^c, d^ | Not mention | **⨁◯◯◯**  **Very low** |
| [Sanaz Malekian](https://pubmed.ncbi.nlm.nih.gov/?sort=pubdate&size=50&term=Malekian+S&cauthor_id=34187123)，2021(Malekian et al., 2021) | TNF-α | Adults | *N. sativa/*Placebo | 5 | 262 | MD | -2.15 | -3.22, -1.09 | 32% | <0.0001 | Serious ^a^ | Not serious | Not serious | Serious ^c^ | Not mention | **⨁⨁◯◯**  **Low** |
|  | hs-CRP | Adults | *N. sativa/*Placebo | 5 | 287 | MD | -0.98 | -1.98, 0.03 | 78% | 0.06 | Serious ^a^ | Very serious ^b^ | Not serious | Very serious ^c, d^ | Not mention | **⨁◯◯◯**  **Very low** |
|  | IL-6 | Adults | *N. sativa/*Placebo | 2 | 134 | MD | -0.25 | -0.65, 0.16 | 0% | 0.23 | Serious ^a^ | Not serious | Not serious | Serious ^c^ | Not mention | **⨁⨁◯◯**  **Low** |
|  | SOD | Adults | *N. sativa/*Placebo | 2 | 88 | MD | 63.79 | 6.84, 120.75 | 0% | 0.03 | Serious ^a^ | Not serious | Not serious | Serious ^c^ | Not mention | **⨁⨁◯◯**  **Low** |
|  | MDA | Adults | *N. sativa/*Placebo | 4 | 179 | MD | -0.95 | -1.97, 0.08 | 68% | 0.07 | Serious ^a^ | Serious ^e^ | Not serious | Very serious ^c, d^ | Not mention | **⨁◯◯◯**  **Very low** |
|  | TAC | Adults | *N. sativa/*Placebo | 4 | 232 | MD | 0.34 | 0.04,0.63 | 94% | 0.03 | Serious ^a^ | Very serious ^b^ | Not serious | Serious ^c^ | Not mention | **⨁◯◯◯**  **Very low** |
| [Rahele Sadat Montazeri](https://pubmed.ncbi.nlm.nih.gov/?sort=pubdate&size=50&term=Montazeri+RS&cauthor_id=33559935)，2021(Montazeri et al., 2021) | hs-CRP | Adults | *N. sativa/*Placebo | 7 | 400 | WMD | -0.67 | -1.29, -0.05 | 95.7% | 0.000 | Serious ^a^ | Very serious ^b^ | Not serious | Not serious | Not serious | **⨁◯◯◯**  **Very low** |
|  | TNF-α | Adults | *N. sativa/*Placebo | 5 | 266 | WMD | -2.29 | -4.48, -0.11 | 93% | 0.000 | Serious ^a^ | Very serious ^b^ | Not serious | Serious ^c^ | Not serious | **⨁◯◯◯**  **Very low** |
|  | MDA | Adults | *N. sativa/*Placebo | 4 | NR | WMD | -1.18 | -2.24, -0.12 | 85.4% | 0.000 | Serious ^a^ | Very serious ^b^ | Not serious | Serious ^c^ | Not serious | **⨁◯◯◯**  **Very low** |
|  | TAC | Adults | *N. sativa/*Placebo | 4 | NR | WMD | 0.35 | 0.10, 0.59 | 77.1% | 0.000 | Serious ^a^ | Very serious ^b^ | Not serious | Serious ^c^ | Serious ^f^ | **⨁◯◯◯**  **Very low** |
|  | SOD | Adults | *N. sativa/*Placebo | 3 | NR | WMD | 66.30 | 1.03, 131.57 | 99.4% | 0.000 | Serious ^a^ | Very serious ^b^ | Not serious | Serious ^c^ | Not serious | **⨁◯◯◯**  **Very low** |
| [Gang Tang](https://pubmed.ncbi.nlm.nih.gov/?sort=pubdate&size=50&term=Tang+G&cauthor_id=33728708)，2021(Tang et al., 2021) | ALT | NAFLD | *N. sativa/*Placebo | 5 | 358 | MD | -11.23 | -21.41, -1.05 | 96% | 0.00001 | Serious ^a^ | Very serious ^b^ | Not serious | Not serious | Not mention | **⨁◯◯◯**  **Very low** |
|  | AST | NAFLD | *N. sativa/*Placebo | 5 | 358 | MD | -12.00 | -19.38, -4.62 | 90% | 0.00001 | Serious ^a^ | Very serious ^b^ | Not serious | Not serious | Not mention | **⨁◯◯◯**  **Very low** |
|  | FBS | NAFLD | *N. sativa/*Placebo | 3 | 168 | MD | -5.53 | -7.39, -3.68 | 0% | 0.53 | Serious ^a^ | Not serious | Not serious | Serious ^c^ | Not mention | **⨁◯◯◯**  **Very low** |
|  | Insulin | NAFLD | *N. sativa/*Placebo | 2 | 87 | MD | -1.1 | -4.29, 2.09; | 80% | 0.03 | Serious ^a^ | Very serious ^b^ | Not serious | Very serious ^c, d^ | Not mention | **⨁◯◯◯**  **Very low** |
|  | TC | NAFLD | *N. sativa/*Placebo | 3 | 168 | MD | -7.97 | -32.87, 16.94 | 90% | 0.0001 | Serious ^a^ | Very serious ^b^ | Not serious | Very serious ^c, d^ | Not mention | **⨁◯◯◯**  **Very low** |
|  | TG | NAFLD | *N. sativa/*Placebo | 4 | 288 | MD | -10.16 | -23.36, 3.04 | 52% | 0.1 | Serious ^a^ | Serious ^e^ | Not serious | Very serious ^c, d^ | Not mention | **⨁◯◯◯**  **Very low** |
|  | HDL | NAFLD | *N. sativa/*Placebo | 4 | 288 | MD | 5.41 | 2.47, 8.34 | 75% | 0.007 | Serious ^a^ | Serious ^e^ | Not serious | Serious ^c^ | Not mention | **⨁◯◯◯**  **Very low** |
|  | LDL | NAFLD | *N. sativa/*Placebo | 4 | 288 | MD | -7.55 | -17.80, 2.70 | 83% | 0.0006 | Serious ^a^ | Very serious ^b^ | Not serious | Very serious ^c, d^ | Not mention | **⨁◯◯◯**  **Very low** |
|  | hs-CRP | NAFLD | *N. sativa/*Placebo | 2 | 87 | MD | -0.35 | -0.65, -0.05; | 0% | 0.4 | Serious ^a^ | Not serious | Not serious | Serious ^c^ | Not mention | **⨁◯◯◯**  **Very low** |
|  | TNF-α | NAFLD | *N. sativa/*Placebo | 2 | 87 | MD | -0.99 | -2.91, 0.92 | 62% | 0.11 | Serious ^a^ | Serious ^e^ | Not serious | Very serious ^c, d^ | Not mention | **⨁◯◯◯**  **Very low** |
|  | Grade of fatty liver | NAFLD | *N. sativa/*Placebo | 3 | 271 | RR | 3.65 | 1.15, 11.57; | 74% | 0.02 | Serious ^a^ | Serious ^e^ | Not serious | Serious ^c^ | Not mention | **⨁◯◯◯**  **Very low** |
| [M Ardiana](https://pubmed.ncbi.nlm.nih.gov/?sort=pubdate&size=50&term=Ardiana+M&cauthor_id=32454800)，2020(Ardiana et al., 2020) | MDA | Adults | *N. sativa/*Placebo | 4 | NR | WMD | -0.532 | -1.192, 0.128 | NR | 0.114 | Not serious | Serious ^e^ | Not serious | Very serious ^c, d^ | Not mention | **⨁◯◯◯**  **Very low** |
|  | SOD | Adults | *N. sativa/*Placebo | 4 | NR | WMD | 48.189 | 30.295,66.083 | NR | 0.01 | Not serious | Serious ^e^ | Not serious | Serious ^c^ | Not mention | **⨁◯◯◯**  **Very low** |
|  | TAC | Adults | *N. sativa/*Placebo | 4 | NR | WMD | 0.219 | -0.136, 0.573 | NR | 0.227 | Not serious | Serious ^e^ | Not serious | Very serious ^c, d^ | Not mention | **⨁◯◯◯**  **Very low** |
| [Jamal Hallajzadeh](https://pubmed.ncbi.nlm.nih.gov/?sort=pubdate&size=50&term=Hallajzadeh+J&cauthor_id=32394508), 2020(Hallajzadeh et al., 2020) | TC | Patients with various disorders | *N. sativa/*Placebo | 34 | NR | WMD | -16.80 | -21.04, -12.55 | 90.9% | 0.000 | Serious ^a^ | Very serious ^b^ | Not serious | Not serious | Not serious | **⨁◯◯◯**  **Very low** |
|  | TG | Patients with various disorders | *N. sativa/*Placebo | 38 | NR | WMD | -15.73 | -20.77, -10.69 | 98.8% | 0.000 | Serious ^a^ | Very serious ^b^ | Not serious | Not serious | Not serious | **⨁◯◯◯**  **Very low** |
|  | LDL-C | Patients with various disorders | *N. sativa/*Placebo | 37 | NR | WMD | -18.45 | -22.44, -14.45 | 96.2 | 0.000 | Serious ^a^ | Very serious ^b^ | Not serious | Not serious | Not serious | **⨁◯◯◯**  **Very low** |
|  | HDL-C | Patients with various disorders | *N. sativa/*Placebo | 39 | NR | WMD | 1.93 | 1.23, 2.64 | 84.1% | 0.000 | Serious ^a^ | Very serious ^b^ | Not serious | Not serious | Not serious | **⨁◯◯◯**  **Very low** |
|  | VLDL | Patients with various disorders | *N. sativa/*Placebo | 4 | NR | WMD | -3.72 | -7.27, -0.18 | 94.9 | 0.000 | Serious ^a^ | Very serious ^b^ | Not serious | Not serious | Serious ^f^ | **⨁◯◯◯**  **Very low** |
|  | FBS | Patients with various disorders | *N. sativa/*Placebo | 29 | NR | WMD | -15.18 | -19.82, -10.55 | 97.2 | 0.000 | Serious ^a^ | Very serious ^b^ | Not serious | Not serious | Not serious | **⨁◯◯◯**  **Very low** |
|  | HbA1C | Patients with various disorders | *N. sativa/*Placebo | 9 | NR | WMD | -0.45 | -0.66, -0.23 | 88.1% | 0.000 | Serious ^a^ | Very serious ^b^ | Not serious | Not serious | Serious ^f^ | **⨁◯◯◯**  **Very low** |
|  | Insulin | Patients with various disorders | *N. sativa/*Placebo | 12 | NR | WMD | 0.92 | -0.25, 2.09 | 88.7% | 0.000 | Serious ^a^ | Very serious ^b^ | Not serious | Serious ^d^ | Not serious | **⨁◯◯◯**  **Very low** |
|  | HOMA-IR | Patients with various disorders | *N. sativa/*Placebo | 4 | NR | WMD | -0.35 | -1.18, 0.47 | 79.4% | 0.002 | Serious ^a^ | Very serious ^b^ | Not serious | Serious ^d^ | Serious ^f^ | **⨁◯◯◯**  **Very low** |
|  | CRP | Patients with various disorders | *N. sativa/*Placebo | 8 | NR | WMD | -3.61 | -9.23, 2.01 | 99.9% | 0.000 | Serious ^a^ | Very serious ^b^ | Not serious | Serious ^d^ | Serious ^f^ | **⨁◯◯◯**  **Very low** |
|  | TNF-α | Patients with various disorders | *N. sativa/*Placebo | 6 | NR | WMD | -1.18 | -3.23, 0.86 | 80.5% | 0.000 | Serious ^a^ | Very serious ^b^ | Not serious | Serious ^d^ | Serious ^f^ | **⨁◯◯◯**  **Very low** |
|  | MDA | Patients with various disorders | *N. sativa/*Placebo | 5 | NR | WMD | -0.95 | -2.18, 0.27 | 92.2% | 0.000 | Serious ^a^ | Very serious ^b^ | Not serious | Serious ^d^ | Serious ^f^ | **⨁◯◯◯**  **Very low** |
|  | TAC | Patients with various disorders | *N. sativa/*Placebo | 5 | NR | WMD | 0.31 | 0.00, 0.63 | 94.3% | 0.000 | Serious ^a^ | Very serious ^b^ | Not serious | Serious ^d^ | Serious ^f^ | **⨁◯◯◯**  **Very low** |
| [Mohsen Mohit](https://pubmed.ncbi.nlm.nih.gov/?sort=pubdate&size=50&term=Mohit+M&cauthor_id=33183658)，2020(Mohit et al., 2020) | CRP | Adults | *N. sativa/*Placebo | 6 | NR | SMD | -0.35 | -0.59, -0.12 | 10.5% | 0.34 | Serious ^a^ | Not serious | Not serious | Not serious | Not serious | **⨁◯◯◯**  **Very low** |
|  | TNF-α | Adults | *N. sativa/*Placebo | 6 | NR | SMD | -0.35 | -0.70, 0.01 | 58.2% | 0.03 | Serious ^a^ | Serious ^e^ | Not serious | Serious ^d^ | Not serious | **⨁◯◯◯**  **Very low** |
|  | MDA | Adults | *N. sativa/*Placebo | 6 | NR | SMD | -0.56 | -0.98, -0.15 | 64.7% | 0.01 | Serious ^a^ | Serious ^e^ | Not serious | Not serious | Not serious | **⨁⨁◯◯**  **Low** |
|  | TAC | Adults | *N. sativa/*Placebo | 6 | NR | SMD | 0.48 | 0.09, 0.87 | 65.6% | 0.01 | Serious ^a^ | Serious ^e^ | Not serious | Not serious | Not serious | **⨁⨁◯◯**  **Low** |
| [Elham Razmpoosh](https://pubmed.ncbi.nlm.nih.gov/?sort=pubdate&size=50&term=Razmpoosh+E&cauthor_id=32201245)，2020(Razmpoosh et al., 2020) | AST | Adults | *N. sativa/*Placebo | 13 | 846 | WMD | -0.539 | -1.715, 0.636 | 86.3% | 0.000 | Serious ^a^ | Very serious ^b^ | Not serious | Serious ^d^ | Not serious | **⨁◯◯◯**  **Very low** |
|  | ALP | Adults | *N. sativa/*Placebo | 9 | 710 | WMD | -10.825 | -19.658, -1.992 | 75.7% | 0.000 | Serious ^a^ | Very serious ^b^ | Not serious | Not serious | Not serious | **⨁◯◯◯**  **Very low** |
|  | ALT | Adults | *N. sativa/*Placebo | 15 | 1119 | WMD | -0.074 | -0.682, 0.534 | 38.4% | 0.065 | Serious ^a^ | Not serious | Not serious | Serious ^d^ | Not serious | **⨁⨁◯◯**  **Low** |
|  | BUN | Adults | *N. sativa/*Placebo | 12 | 821 | WMD | -1.016 | -1.760, -0.273 | 87.7% | 0.000 | Serious ^a^ | Very serious ^b^ | Not serious | Not serious | Not serious | **⨁◯◯◯**  **Very low** |
|  | CREA | Adults | *N. sativa/*Placebo | 15 | 998 | WMD | -2.300 | -4.678, 0.078 | 86.1% | 0.000 | Serious ^a^ | Very serious ^b^ | Not serious | Serious ^d^ | Not serious | **⨁◯◯◯**  **Very low** |
|  | Bilirubin | Adults | *N. sativa/*Placebo | 5 | NR | WMD | 0.004 | -0.023, 0.030 | 21.7% | 0.786 | Serious ^a^ | Not serious | Not serious | Serious ^d^ | Not serious | **⨁⨁◯◯**  **Low** |
|  | Uric acid | Adults | *N. sativa/*Placebo | 4 | NR | WMD | 0.105 | -0.001, 0.212 | 0% | 0.053 | Serious ^a^ | Not serious | Not serious | Serious ^d^ | Not serious | **⨁⨁◯◯**  **Low** |
| [Rahele Tavakoly](https://pubmed.ncbi.nlm.nih.gov/?sort=pubdate&size=50&term=Tavakoly+R&cauthor_id=31331553)，2019(Tavakoly et al., 2019) | CRP | Adults | *N. sativa/*Placebo | 5 | 348 | WMD | -0.55 | -1.02, -0.08 | 77.3% | 0.001 | Serious ^a^ | Very serious ^b^ | Serious ^g^ | Not serious | Serious ^f^ | **⨁◯◯◯**  **Very low** |
| [Seyed Mohammad Mousavi](https://pubmed.ncbi.nlm.nih.gov/?sort=pubdate&size=50&term=Mousavi+SM&cauthor_id=29857879)，2018(Mousavi et al., 2018) | BW | Adults | *N. sativa/*Placebo | 10 | NR | WMD | -1.76 | -3.34, -0.17 | 87.4% | 0.02 | Serious ^a^ | Very serious ^b^ | Not serious | Not serious | Not serious | **⨁◯◯◯**  **Very low** |
|  | BMI | Adults | *N. sativa/*Placebo | 11 | NR | WMD | -0.85 | -1.23, -0.46 | 70.6% | <0.001 | Serious ^a^ | Serious ^e^ | Not serious | Not serious | Not serious | **⨁⨁◯◯**  **Low** |
|  | WC | Adults | *N. sativa/*Placebo | 5 | NR | WMD | -4.04 | -11.38, 3.27 | 97.8% | 0.27 | Serious ^a^ | Very serious ^b^ | Not serious | Serious ^d^ | Serious ^f^ | **⨁◯◯◯**  **Very low** |
| [Nazli Namazi](https://pubmed.ncbi.nlm.nih.gov/?sort=pubdate&size=50&term=Namazi+N&cauthor_id=29559374)，2018(Namazi et al., 2018) | BW | Adults | *N. sativa/*Placebo | 7 | NR | WMD | -2.11 | -3.61, -0.61 | 72.4% | 0.001 | Serious ^a^ | Serious ^e^ | Not serious | Not serious | Not serious | **⨁⨁◯◯**  **Low** |
|  | BMI | Adults | *N. sativa/*Placebo | 9 | NR | WMD | -1.16 | -1.81, -0.51 | 40.1% | 0.1 | Serious ^a^ | Not serious | Not serious | Not serious | Not serious | **⨁⨁⨁◯**  **Moderate** |
|  | WC | Adults | *N. sativa/*Placebo | 5 | NR | WMD | -6.46 | -9.85, -3.06 | 98.2% | 0.0001 | Serious ^a^ | Very serious ^b^ | Not serious | Not serious | Not serious | **⨁◯◯◯**  **Very low** |
| [Reza Daryabeygi-Khotbehsara](https://pubmed.ncbi.nlm.nih.gov/?sort=pubdate&size=50&term=Daryabeygi-Khotbehsara+R&cauthor_id=29154069)，2017(Daryabeygi-Khotbehsara et al., 2017) | FBS | T2DM | *N. sativa/*Placebo and standard treatment | 5 | NR | WMD | -17.84 | -21.19, -14.49 | 28.5% | 0.23 | Serious ^a^ | Not serious | Not serious | Not serious | Not serious | **⨁⨁⨁◯**  **Moderate** |
|  | HbA1c | T2DM | *N. sativa/*Placebo and standard treatment | 5 | NR | WMD | -0.71 | -1.04, -0.39 | 89.3% | 0.000 | Serious ^a^ | Very serious ^b^ | Not serious | Not serious | Not serious | **⨁◯◯◯**  **Very low** |
|  | TC | T2DM | *N. sativa/*Placebo and standard treatment | 4 | NR | WMD | -22.99 | -32.16, -13.83 | 31.9% | 0.22 | Serious ^a^ | Not serious | Not serious | Not serious | Not serious | **⨁⨁⨁◯**  **Moderate** |
|  | TG | T2DM | *N. sativa/*Placebo and standard treatment | 4 | NR | WMD | -6.80 | -33.59, 19.99 | 89.5% | 0.001 | Serious ^a^ | Very serious ^b^ | Not serious | Serious ^d^ | Not serious | **⨁◯◯◯**  **Very low** |
|  | HDL-C | T2DM | *N. sativa/*Placebo and standard treatment | 5 | NR | WMD | 0.37 | -1.59, 2.33 | 0.0% | 0.52 | Serious ^a^ | Not serious | Not serious | Serious ^d^ | Not serious | **⨁⨁◯◯**  **Low** |
|  | LDL-C | T2DM | *N. sativa/*Placebo and standard treatment | 5 | NR | WMD | -22.38 | -33.60, -11.15 | 80.0% | 0.01 | Serious ^a^ | Very serious ^b^ | Not serious | Not serious | Not serious | **⨁◯◯◯**  **Very low** |
| [Amirhossein Sahebkar](https://pubmed.ncbi.nlm.nih.gov/?sort=pubdate&size=50&term=Sahebkar+A&cauthor_id=26875640)，2016(Sahebkar et al., 2016a) | TC | Adults | *N. sativa/*Placebo | 15 | NR | WMD | -15.65 | -24.67, -6.63 | NR | NR | Serious ^a^ | Serious ^e^ | Not serious | Not serious | Not serious | **⨁⨁◯◯**  **Low** |
|  | LDL-C | Adults | *N. sativa/*Placebo | 16 | NR | WMD | -14.10 | -19.32, -8.88 | NR | NR | Serious ^a^ | Serious ^e^ | Not serious | Not serious | Serious ^f^ | **⨁◯◯◯**  **Very low** |
|  | TG | Adults | *N. sativa/*Placebo | 16 | NR | WMD | -20.64 | -30.29, -11.00 | NR | NR | Serious ^a^ | Serious ^e^ | Not serious | Not serious | Serious ^f^ | **⨁◯◯◯**  **Very low** |
|  | HDL-C | Adults | *N. sativa/*Placebo | 18 | NR | WMD | 0.28 | -1.96, 2.53 | NR | NR | Serious ^a^ | Serious ^e^ | Not serious | Not serious | Serious ^f^ | **⨁◯◯◯**  **Very low** |
| [Amirhossein Sahebkar](https://pubmed.ncbi.nlm.nih.gov/?sort=pubdate&size=50&term=Sahebkar+A&cauthor_id=27512971) ，2016(Sahebkar et al., 2016b) | SBP | Patients with normal or high blood pressure | *N. sativa/*Placebo and standard treatment | 11 | 860 | WMD | -3.26 | -5.10, -1.42, | 59% | 0.07 | Serious ^a^ | Serious ^e^ | Not serious | Not serious | Not serious | **⨁◯◯◯**  **Very low** |
|  | DBP | Patients with normal or high blood pressure | *N. sativa/*Placebo and standard treatment | 11 | 860 | WMD | -2.80 | -4.28, -1.32, | 60% | 0.07 | Serious ^a^ | Serious ^e^ | Not serious | Not serious | Not serious | **⨁⨁◯◯**  **Low** |

Abbreviations: MD: mean difference；SMD standard mean difference；RR: relative risk; OR: odd risk; NR：not reported; I: intervention; C: comparator; NAFLD, non-alcoholic fatty liver diseases; T2DM, type 2 diabetes mellitus; BMI: Body Mass Index; FPG: fasting plasma glucose; OGTT: oral glucose tolerance test; HbA1c: hemoglobin A1c; HOMA-IR: homeostatic model assessment of insulin resistance; TG: triglyceride; TC: total cholesterol; LDL-C: low-density lipoprotein cholesterol; HDL-C: high-density lipoprotein cholesterol; CRP: C-reactive protein; CREA: Creatinine; MDA: malondialdehyde; ALT: alanine aminotransferase; AST: aspartate aminotransferase; FBG: fasting blood glucose; PPBG: postprandial blood glucose; BW: body weight；ACT: asthma control test; FEV1: forced expiratory volume at 1s; PEF: peak expiratory flow; IL-4: interlukin-4; IFN-γ: interferon γ; TNF-α: tumor necrosis factor-α; hs-CRP: high-sensitive C-Reactive Protein; IL-6: interlukin-6; SOD: superoxide dismutase; TAC: total antioxidant capacity; VLDL: very low-density lipoproteins; BUN: blood urea nitrogen; SBP: systolic blood pressure; DBP: diastolic blood pressure. ◯ represented “low”; ⨁ represented “high”.

^a^ The included study had an unclear risk of selection, performance, detection, and reporting biases; ^b^ I^2^ ≥ 75%; ^c^ Sample size <300; ^d^ 95 % Cl includes invalid line; ^e^ 50% ≤ I^2^ < 75%; ^f^ Funnel plot or Egger's or Begg's tests indicated asymmetry; ^g^ Inconsistent measuring indicators

References:

Ardiana, M., Pikir, B.S., Santoso, A., Hermawan, H.O., and Al-Farabi, M.J. (2020). Effect of Supplementation on Oxidative Stress and Antioxidant Parameters: A Meta-Analysis of Randomized Controlled Trials. *TheScientificWorldJournal* 2020**,** 2390706. doi: 10.1155/2020/2390706.

Azizi, N., Amini, M.R., Djafarian, K., and Shab-Bidar, S. (2021). The Effects of Supplementation on Liver Enzymes Levels: a Systematic Review and Meta-analysis of Randomized Controlled Trials. *Clinical Nutrition Research* 10(1)**,** 72-82. doi: 10.7762/cnr.2021.10.1.72.

Chattopadhyay, K., Wang, H., Kaur, J., Nalbant, G., Almaqhawi, A., Kundakci, B., et al. (2022). Effectiveness and Safety of Ayurvedic Medicines in Type 2 Diabetes Mellitus Management: A Systematic Review and Meta-Analysis. *Frontiers In Pharmacology* 13**,** 821810. doi: 10.3389/fphar.2022.821810.

Daryabeygi-Khotbehsara, R., Golzarand, M., Ghaffari, M.P., and Djafarian, K. (2017). Nigella sativa improves glucose homeostasis and serum lipids in type 2 diabetes: A systematic review and meta-analysis. *Complementary Therapies In Medicine* 35. doi: 10.1016/j.ctim.2017.08.016.

Golpour-Hamedani, S., Hadi, A., SafariMalekabadi, D., Najafgholizadeh, A., Askari, G., and Pourmasoumi, M. (2022). The effect of nigella supplementation on blood pressure: A systematic review and dose-response meta-analysis. *Critical Reviews In Food Science and Nutrition*. doi: 10.1080/10408398.2022.2110566.

Gyawali, D., Vohra, R., Orme-Johnson, D., Ramaratnam, S., and Schneider, R.H. (2021). A Systematic Review and Meta-Analysis of Ayurvedic Herbal Preparations for Hypercholesterolemia. *Medicina (Kaunas, Lithuania)* 57(6)**,** 546. doi: 10.3390/medicina57060546.

Hallajzadeh, J., Milajerdi, A., Mobini, M., Amirani, E., Azizi, S., Nikkhah, E., et al. (2020). Effects of Nigella sativa on glycemic control, lipid profiles, and biomarkers of inflammatory and oxidative stress: A systematic review and meta-analysis of randomized controlled clinical trials. *Phytotherapy Research : PTR* 34(10)**,** 2586-2608. doi: 10.1002/ptr.6708.

Han, A., and Shi, D. (2021). The efficacy of Nigella sativa supplementation for asthma control: a meta-analysis of randomized controlled studies. *Postepy Dermatologii I Alergologii* 38(4)**,** 561-565. doi: 10.5114/ada.2020.93220.

Malekian, S., Ghassab-Abdollahi, N., Mirghafourvand, M., and Farshbaf-Khalili, A. (2021). The effect of Nigella Sativa on oxidative stress and inflammatory biomarkers: a systematic review and meta-analysis. *Journal of Complementary & Integrative Medicine* 18(2)**,** 235-259. doi: 10.1515/jcim-2019-0198.

Mohit, M., Farrokhzad, A., Faraji, S.N., Heidarzadeh-Esfahani, N., and Kafeshani, M. (2020). Effect of Nigella sativa L. supplementation on inflammatory and oxidative stress indicators: A systematic review and meta-analysis of controlled clinical trials. *Complementary Therapies In Medicine* 54**,** 102535. doi: 10.1016/j.ctim.2020.102535.

Montazeri, R.S., Fatahi, S., Sohouli, M.H., Abu-Zaid, A., Santos, H.O., Găman, M.-A., et al. (2021). The effect of nigella sativa on biomarkers of inflammation and oxidative stress: A systematic review and meta-analysis of randomized controlled trials. *Journal of Food Biochemistry* 45(4)**,** e13625. doi: 10.1111/jfbc.13625.

Mousavi, S.M., Sheikhi, A., Varkaneh, H.K., Zarezadeh, M., Rahmani, J., and Milajerdi, A. (2018). Effect of Nigella sativa supplementation on obesity indices: A systematic review and meta-analysis of randomized controlled trials. *Complementary Therapies In Medicine* 38**,** 48-57. doi: 10.1016/j.ctim.2018.04.003.

Namazi, N., Larijani, B., Ayati, M.H., and Abdollahi, M. (2018). The effects of Nigella sativa L. on obesity: A systematic review and meta-analysis. *Journal of Ethnopharmacology* 219**,** 173-181. doi: 10.1016/j.jep.2018.03.001.

Razmpoosh, E., Safi, S., Abdollahi, N., Nadjarzadeh, A., Nazari, M., Fallahzadeh, H., et al. (2020). The effect of Nigella sativa on the measures of liver and kidney parameters: A systematic review and meta-analysis of randomized-controlled trials. *Pharmacological Research* 156**,** 104767. doi: 10.1016/j.phrs.2020.104767.

Saadati, S., Naseri, K., Asbaghi, O., Abhari, K., Zhang, P., Li, H.-B., et al. (2022). Nigella sativa supplementation improves cardiometabolic indicators in population with prediabetes and type 2 diabetes mellitus: A systematic review and meta-analysis of randomized controlled trials. *Frontiers In Nutrition* 9**,** 977756. doi: 10.3389/fnut.2022.977756.

Sahebkar, A., Beccuti, G., Simental-Mendía, L.E., Nobili, V., and Bo, S. (2016a). Nigella sativa (black seed) effects on plasma lipid concentrations in humans: A systematic review and meta-analysis of randomized placebo-controlled trials. *Pharmacological Research* 106**,** 37-50. doi: 10.1016/j.phrs.2016.02.008.

Sahebkar, A., Soranna, D., Liu, X., Thomopoulos, C., Simental-Mendia, L.E., Derosa, G., et al. (2016b). A systematic review and meta-analysis of randomized controlled trials investigating the effects of supplementation with Nigella sativa (black seed) on blood pressure. *Journal of Hypertension* 34(11)**,** 2127-2135. doi: 10.1097/HJH.0000000000001049.

Tang, G., Zhang, L., Tao, J., and Wei, Z. (2021). Effect of Nigella sativa in the treatment of nonalcoholic fatty liver disease: A systematic review and meta-analysis of randomized controlled trials. *Phytotherapy Research : PTR* 35(8)**,** 4183-4193. doi: 10.1002/ptr.7080.

Tavakoly, R., Arab, A., Vallianou, N., Clark, C.C.T., Hadi, A., Ghaedi, E., et al. (2019). The effect of Nigella sativa L. supplementation on serum C-reactive protein: A systematic review and meta-analysis of randomized controlled trials. *Complementary Therapies In Medicine* 45**,** 149-155. doi: 10.1016/j.ctim.2019.06.008.

Tiwari, A., G, S., Meka, S., Varghese, B., Vishwakarma, G., and Adela, R. (2022). The effect of Nigella sativa on non-alcoholic fatty liver disease: A systematic review and meta-analysis. *Human Nutrition and Metabolism* 28. doi: 10.1016/j.hnm.2022.200146.
